# Association of obesity and lipid indexes with rapid kidney function decline and the progression to chronic kidney disease: a study from a large longitudinal cohort among middle-aged and older adults in China

**DOI:** 10.3389/fmed.2026.1816603

**Published:** 2026-05-04

**Authors:** Wen-Ze Jiang, Meng-Ting Zhu, Jiao-Jiao Hu, Meng-Li Xu, Ke-Da Lu

**Affiliations:** 1 Department of Nephrology, The Third Affiliated Hospital of Zhejiang Chinese Medical University, Hangzhou, China; 2 The Third Clinical Medical School, Zhejiang Chinese Medical University, Hangzhou, China

**Keywords:** CHARLS, chronic kidney disease, lipid, obesity, rapid kidney function decline

## Abstract

**Background:**

Obesity and lipid abnormalities are recognized as important risk factors for rapid kidney function decline (RKFD) and chronic kidney disease (CKD). However, studies on the association between obesity and lipid indices with RKFD and CKD are still lacking. This study aims to investigate the association between obesity and lipid indexes with RKFD and CKD, and further explore their predictive value.

**Methods:**

Data from the China Health and Retirement Longitudinal Study (CHARLS) were used in this study. The obesity and lipid indexes used in this study included non-invasive anthropometric indexes and invasive anthropometric indexes. Multivariate logistic regression models with covariate adjustment were employed to assess association between obesity and lipid indexes and RKFD or progression to CKD. Restricted cubic spline (RCS) regression analyses were performed to characterize potential nonlinear relationships. Predictive performance was quantified through receiver operating characteristic (ROC) curve analysis. Subgroup analysis was performed based on hypertension, diabetes, or cardiovascular disease (CVD) status of the participants.

**Results:**

A total of 3,829 participants were included in this study. Of these, 192 participants developed RKFD and 60 progressed to CKD. Logistic regression after adjusting for confounders revealed significant associations between 11 indexes and RKFD, and 4 indexes standard deviation increases associated with CKD. RCS curve analysis demonstrated that 9 indexes had linear relationship with the risk of progression to RKFD though WHtR, BRI and TyG-BRI had non-linear relationship. Moreover, LAP had a linear relationship with the risk of CKD, whereas VAI had a nonlinear relationship. ROC analysis revealed TyG as the superior RKFD predictor and CVAI as the optimal CKD progression indicator. In subgroup analysis, the association between partial indexes and progression to CKD was more significant in subjects with hypertension (TyG-CVAI) or without CVD (C-index, RFM, TyG-RFM).

**Conclusion:**

This study comprehensively analyzed the associations between 20 obesity and lipid indexes and both RKFD and CKD. We proved that multiple obesity and lipid indexes were associated with RKFD and CKD. Compared with non-invasive anthropometric indexes, invasive anthropometric indexes have a more significant association with RKFD and CKD and have higher predictive value.

## Introduction

Chronic kidney disease (CKD) is a progressive disease characterized by structural and functional impairment of the kidneys, defined as estimated glomerular filtration rate (eGFR) below 60 mL/min/1.73m^2^ persisting for at least three months (1). As the global population ages, CKD has emerged as a serious public health challenge. CKD is expected to be the fifth leading cause of death globally by 2040 and one of the largest expected growth among all major causes of death (2). Early detection and intervention are effective ways to prevent the progression of CKD. Therefore, finding accurate and convenient indexes to predict rapid decline in kidney function is a key priority in the field of nephrology.

Obesity is a global health issue that commonly affects middle-aged and older individuals, and it raises the risk of several chronic conditions, including hypertension, diabetes, and cardiovascular diseases (CVD) (3–5). Existing studies have highlighted the close relationship between obesity and CKD (6). The impact of body mass index (BMI), waist circumference (WC) and waist-to-height ratio (WHtR) on the risk of CKD has been reported by several studies (7–9). However, BMI does not distinguish between muscle and fat, which is only a surrogate indicator of body obesity. While WC effectively reflects body size, fat percentage, and distribution, its strong association with BMI complicates the distinction between their respective contributions as separate epidemiological risk factors (10). Moreover, the negative impact of lipid abnormalities on the kidney is gaining more attention (11). Therefore, many new obesity and lipid indexes, including a body shape index (ABSI), body roundness index (BRI), conicity index (C-index), relative fat mass (RFM), weight-adjusted waist index (WWI), visceral adiposity index (VAI), Chinese visceral adiposity index (CVAI), lipid accumulation product (LAP), and triglyceride glucose (TyG) index have been proposed to use in research (12–18).

To more comprehensively assess the association between obesity and kidney function rapid decline (RKFD), as well as CKD, we conducted a longitudinal cohort study based on the Chinese Longitudinal Study of Health and Retirement (CHARLS). The purpose of this study is to explore the relationship between 20 obesity and lipid indexes and RKFD or progression to CKD in middle-aged and elderly Chinese people with normal renal function. We hope to find effective indexes for predicting the rapid decline of kidney function, which will provide an important reference for research work in the field of obesity and kidney disease.

## Methods

### Study population

The data used in this study were downloaded from CHARLS since 2011 to 2015. CHARLS is a long-term follow-up survey that collects household and personal data on middle-aged and elderly people aged 45 and over in China, aiming to analyze population aging (19). The CHARLS National Baseline Survey was conducted in 2011, followed up in 2013, 2015, 2018, and 2020, and blood specimens were only collected in 2011 and 2015. All the blood samples were well preserved and delivered to a professional testing organization (Youanmen Center for Clinical Laboratory of Capital Medical University). CHARLS was permitted by the Biomedical Ethics Review Committee of Peking University, Beijing, China (IRB00001052–11015). All participants have signed an informed consent form before the entire experiment. All the data and research materials are available at the CHARLS project website.^1^

**Figure 1:**
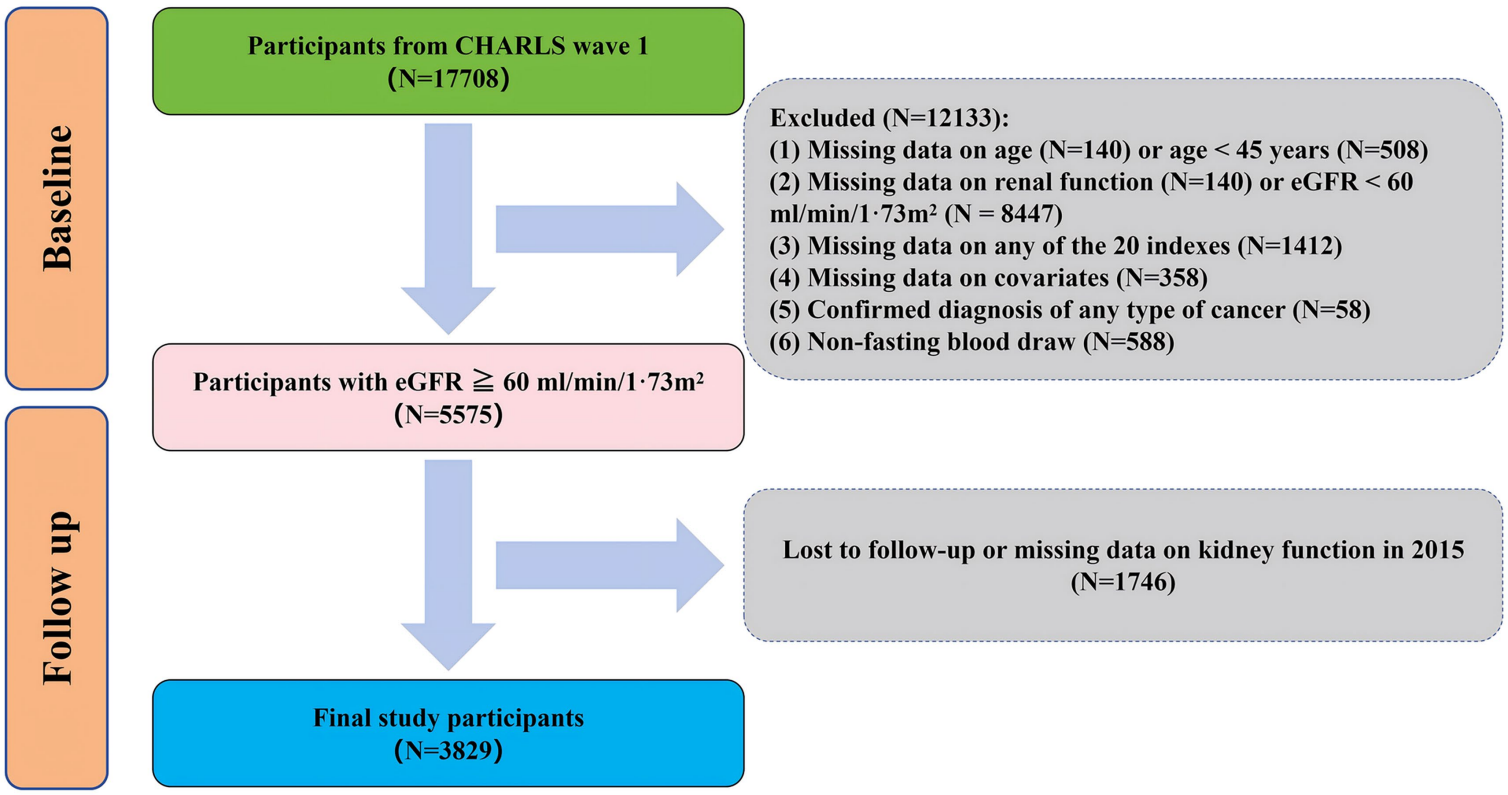
Flow chart of inclusion and exclusion criteria of participants.

We used data from 17,708 participants from CHARLS wave 1. We excluded individuals who met any of the following criteria at baseline: (1) missing data on age (*n* = 140) or age < 45 years (*n* = 508); (2) missing data on renal function (*n* = 140) or eGFR < 60 mL/min/1·73m^2^ (*n* = 8,447); (3) missing data on any of the 20 indexes (*n* = 1,412); (4) missing data on covariates (*n* = 358); (5) confirmed diagnosis of any type of cancer (*n* = 58); (6) non-fasting blood draw (*n* = 588). Of the 5,575 participants included at baseline, 1746 were lost to follow-up or missing data on renal function in 2015. Moreover, we excluded 1746 participants who were lost to follow-up or missing data on kidney function in 2015. Finally, a total of 3,829 participants were used in this study (Figure 1).

### Anthropometric measurements

The anthropometric measurements used in this study included non-invasive anthropometric indexes (including BMI, WC, WHtR, ABSI, BRI, C-index, RFM and WWI) and invasive anthropometric indexes (including VAI, CVAI, LAP, TyG, TyG-BMI (triglyceride glucose × body mass index), TyG-WC (triglyceride glucose × waist circumference), TyG-WHtR (triglyceride glucose × waist-to-height ratio), TyG-ABSI (triglyceride glucose × a body shape index), TyG-BRI (triglyceride glucose × body roundness index), TyG-RFM (triglyceride glucose × relative fat mass), TyG-WWI (triglyceride glucose × weight-adjusted waist index) and TyG-CVAI (triglyceride glucose × Chinese visceral adiposity index)). WC was measured between the iliac crest and the lower ribs on both sides, at the end of expiratory breath. Other anthropometric measurements are calculated using the following equations (Equations 1− 19). It should be noted that invasive anthropometric indexes require blood sampling to evaluate triglyceride (TG) and high-density lipoprotein cholesterol (HDL-C) levels.


$$\mathrm{BMI}=\frac{\text{Weight}(\mathrm{kg})}{{\text{Height}}^{2}(m)}$$ (1)

$$\text{WHtR}=\frac{\mathrm{WC}(\mathrm{cm})}{\text{Height}(\mathrm{cm})}$$ (2)

$$\text{ABSI}=\frac{\mathrm{WC}(m)}{\text{Height}{\left(m\right)}^{1/2}\times {\mathrm{BMI}}^{2/3}}$$ (3)

$$\mathrm{BRI}=364.2-365.5\sqrt{1-[\frac{{\left(\mathrm{WC}(m)/2\pi \right)}^{2}}{{\left(0.5\times \text{Height}(m)\right)}^{2}}]}$$ (4)

$$C-\text{index}=\frac{\mathrm{WC}(m)}{0.109\sqrt{\frac{\text{Weight}(\mathrm{kg})}{\text{Height}(m)}}}$$ (5)

$$\begin{array}{l} \mathrm{RFM}\\ \text{Females}:\mathrm{RFM}=64-(20\times \frac{\text{Height}(\mathrm{cm})}{\mathrm{WC}(\mathrm{cm})})+12\\ \text{Males}:\mathrm{RFM}=64-(20\times \frac{\text{Height}(\mathrm{cm})}{\mathrm{WC}(\mathrm{cm})}) \end{array}$$ (6)

$$\mathrm{WWI}=\frac{\mathrm{WC}(\mathrm{cm})}{\sqrt{\text{Weight}(\mathrm{kg})}}$$ (7)

$$\begin{array}{l} \mathrm{VAI}\\ \begin{array}{l} \text{Females}:\mathrm{VAI}=\frac{\mathrm{WC}(\mathrm{cm})}{36.58+(1.89\times \mathrm{BMI})}\\ +\frac{\mathrm{TG}(\text{mmol}/L)}{0.81}+\frac{1.52}{\mathrm{HDL}-C(\text{mmol}/L)} \end{array}\\ \begin{array}{l} \text{Males}:\mathrm{VAI}=\frac{\mathrm{WC}(\mathrm{cm})}{39.68+(1.88\times \mathrm{BMI})}\\ +\frac{\mathrm{TG}(\text{mmol}/L)}{1.03}+\frac{1.31}{\mathrm{HDL}-C(\text{mmol}/L)} \end{array} \end{array}$$ (8)

$$\begin{array}{l} \text{CVAI}\\ \begin{array}{l} \text{Females}:\text{CVAI}=-187.32+1.71\times \mathrm{age}+4.23\times \mathrm{BMI}+1.12\\ \times \mathrm{WC}(\mathrm{cm})+39.76\times \log_{10}\;\mathrm{TG}(\text{mmol}/L)\\ -11.66\times \mathrm{HDL}-C(\text{mmol}/L) \end{array}\\ \begin{array}{l} \text{Males}:\text{CVAI}=-267.93+0.68+\mathrm{age}+0.03\times \mathrm{BMI}+4.00\\ \times \mathrm{WC}(\mathrm{cm})+22.00\times \log_{10}\mathrm{TG}(\text{mmol}/L)\\ -16.32\times \mathrm{HDL}-C(\text{mmol}/L) \end{array} \end{array}$$ (9)

$$\begin{array}{l} \mathrm{LAP}\;\\ \text{Females}:\mathrm{LAP}=[(\mathrm{WC}(\mathrm{cm})-58)\times \mathrm{TG}\;(\frac{\text{mmol}}{L})]\\ \text{Males}:\mathrm{LAP}=[(\mathrm{WC}(\mathrm{cm})-65)\times \mathrm{TG}\;(\frac{\text{mmol}}{L})] \end{array}$$ (10)

$$\mathrm{TyG}=\ln\frac{\mathrm{TG}(\mathrm{mg}/\mathrm{dl})\times \mathrm{FPG}(\mathrm{mg}/\mathrm{dl})}{2}$$ (11)

TyG−BMI=TyG×BMI (12)

TyG−WC=TyG×WC(cm) (13)

TyG−WHtR=TyG×WHtR (14)

TyG−ABSI=TyG×ABSI (15)

TyG−BRI=TyG×BRI (16)

TyG−RFM=TyG×RFM (17)

TyG−WWI=TyG×WWI (18)

TyG−CVAI=TyG×CVAI (19)

BMI was classified into four groups according to the Chinese obesity standard: thin (BMI < 18.5 kg/m2), normal weight (18.5 ≤ BMI < 24.0 kg/m^2^), overweight (24.0 ≤ BMI < 28.0 kg/m^2^), and obesity (BMI ≥ 28.0 kg/m^2^) (20). Abdominal obesity (AO) was determined according to the following criteria: female (WC ≥ 80 cm) and male (WC ≥ 90 cm) (21). Other indexes are classified into Q1-Q4 based on quartiles.

### Definitions of RKFD and CKD

The combined creatinine-cystatin C equation is used to calculate eGFR (Supplementary Figure S1), which can reduce the influence of pathological conditions and muscle status in middle-aged and elderly people (22). RKFD was defined as eGFR decline of >5 mL/min/1.73m^2^ per year (23). Progression to CKD was defined as eGFR < 60 mL/min/1.73 m^2^ in 2015.

### Definition of diabetes and hypertension

Diabetes was diagnosed based on the following conditions: (1) fasting blood glucose (FBG) > 126 mg/dL or Glycosylated Hemoglobin, Type A1C (HbA1c) > 6.5%, (2) self-reported history of diabetes, or (3) self-reported intake of any medications for diabetes. Hypertension was diagnosed based on the following conditions: (1) the mean blood pressure value of three measurements ≥130 mmHg for systolic blood pressure (SBP) or ≥80 mmHg for diastolic blood pressure (DBP), (2) self-reported history of hypertension, or (3) self-reported intake of any medications for hypertension.

### Covariates

For demographic variables, gender (male and female), age, residence (city and country), education (below high school, high school, and college or above), marital status (married and others), smoking status (never, former and current), drinking status (never, former and current), and residence (rural and urban) were obtained from the interview by standardized questionnaires. Moreover, data on blood biomarkers (FBG, HbA1c, total cholesterol (TC), uric acid (UA), and c reactive protein (CRP)) and the treatment of baseline illnesses (kidney disease, diabetes, hypertension, dyslipidemia and CVD (including heart disease and stroke)) were gathered.

### Statistical analysis

Exposure and covariate data from each participant’s baseline examination were included in the analysis. Classified variables were represented by percentages, and continuous variables were represented by means and standard deviation (SD). Comparisons of the baseline characteristics were performed with Kruskal–Wallis rank sum tests for continuous variables and Chi-square tests for categorical variables. Odds ratio (OR) and 95% confidence interval (CI) for the association of 20 indexes with the occurrence of RKFD or progression to CKD were examined by logistic regression analysis. Based on the crude model, we established three models to explore the relationship between 20 indexes and RKFD or progression to CKD. Model 1 adjusted for gender and age; model 2 further adjusted for education, residence, marital status, drinking status, and smoking status; model 3 further adjusted for SBP, DBP, TC, HbA1c, UA, CRP, baseline eGFR and medication usage (for kidney disease, diabetes, hypertension, dyslipidemia, CVD). The results were presented with ORs and 95% CIs. Moreover, restricted cubic spline (RCS) was used to characterize the nonlinear relationship between obesity and lipid indexes and RKFD or progression to CKD. Finally, to prove the predictive performance of 20 indexes for RKFD or progression to CKD, the area under the receiver operating characteristic (ROC) curve (AUC) value was calculated. We analyzed nonlinear relationships by using restricted cubic spline. To clarify the significance of 20 indexes in different populations, we further investigated the subgroups stratified by hypertension (with or without), diabetes (with or without) and CVD (with or without).

All statistical analysis was performed with SPSS 26.0 and R Vision. Stata 18 and Graphpad Pism 9.5 were used for data analysis. In all situations, *p* < 0.05 was considered statistically significant.

## Result

### Baseline characteristics of the participants

The baseline characteristics of all the participants are demonstrated in Table 1. A total of 3,829 eligible participants were included at baseline, including 192 diagnosed with RKFD and 60 who progressed to CKD. The average age of participants was 59.53 ± 8.87 years, with 2,063 (53.88%) female and 1,766 (46.12%) male.

**Table 1:** The baseline characteristics of study population.

Variable	Total	Non RKFD	RKFD	*p*	Non CKD	CKD	*p*
*N*	3,829	3,637	192		3,732	97	
Gender							
Female (%)	2063 (53.88)	1953 (53.70)	110 (57.29)	0.330	2009 (53.83)	54 (55.67)	0.720
Male (%)	1766 (46.12)	1,684 (46.30)	82 (42.71)		1723 (46.17)	43 (44.33)	
Age (years)	59.53 ± 8.87	59.43 ± 8.82	61.36 ± 9.56	**0.008**	59.31 ± 8.79	68.07 ± 7.76	**<0.001**
Weight (kg)	58.82 ± 11.13	58.73 ± 11.16	60.39 ± 10.44	**0.018**	58.80 ± 11.11	59.64 ± 11.90	0.590
Height (m)	1.58 ± 0.09	1.58 ± 0.09	1.57 ± 0.08	0.165	1.58 ± 0.09	1.57 ± 0.08	0.303
SBP (mmHg)	130.14 ± 21.13	129.96 ± 21.11	133.58 ± 21.12	**0.008**	129.89 ± 21.09	139.51 ± 20.55	**<0.001**
DBP (mmHg)	75.45 ± 11.90	75.42 ± 11.93	76.08 ± 11.40	0.413	75.42 ± 11.89	76.52 ± 12.21	0.313
Follow up years (years)	4.00 ± 0.08	4.00 ± 0.08	4.00 ± 0.10	0.566	4.00 ± 0.08	4.01 ± 0.10	0.411
Education							
Below high school (%)	3,511 (91.69)	3,326 (91.45)	185(96.35)	**0.033**	3,417 (91.56)	94 (96.91)	0.158
High school (%)	284 (7.42)	279 (7.67)	5(2.60)		281 (7.53)	3 (3.09)	
College or above (%)	34 (0.89)	32 (0.88)	2(1.04)		34 (0.91)	0 (0.00)	
Marital status							
Married (%)	3,245 (84.75)	3,093 (85.04)	152 (79.17)	**0.027**	3,163 (84.75)	82 (84.54)	0.953
Others (%)	584 (15.25)	544 (14.96)	40 (20.83)		569 (15.25)	15 (15.46)	
Smoking status							
Current (%)	1,153 (30.11)	1,103 (30.33)	50 (26.04)	0.451	1,124 (30.12)	29 (29.90)	0.998
Former (%)	320 (8.36)	303 (8.33)	17 (8.85)		312 (8.36)	8 (8.25)	
Never (%)	2,356 (61.53)	2,231 (61.34)	125 (65.10)		2,296 (61.52)	60 (61.86)	
Drinking status							
Current (%)	1,249 (32.62)	1,193 (32.80)	56 (29.17)	0.562	1,231 (32.98)	18 (18.56)	**0.008**
Former (%)	320 (8.36)	304 (8.36)	16 (8.33)		308 (8.25)	12 (12.37)	
Never (%)	2,260 (59.02)	2,140 (58.84)	120 (62.50)		2,193 (58.76)	67 (69.07)	
Residence							
Rural (%)	2,560 (66.86)	2,432 (66.87)	128 (66.67)	0.954	2,500 (66.00)	60 (61.86)	0.289
Urban (%)	1,269 (33.14)	1,205 (33.13)	64 (33.33)		1,232 (33.01)	37 (38.14)	
Medication taken for kidney disease (%)							
Yes	106 (2.77)	103 (2.83)	3 (1.56)	0.296	103 (2.76)	3 (3.09)	0.750
No	3,723 (97.23)	3,534 (97.17)	189 (98.44)		3,629 (97.24)	94 (96.91)	
Medication taken for diabetes (%)							
Yes	156 (4.07)	140 (3.85)	16 (8.33)	**0.002**	147 (3.94)	9 (9.28)	**0.009**
No	3,673 (95.93)	3,497 (96.15)	176 (91.67)		3,595 (96.33)	88 (90.72)	
Medication taken for hypertension (%)							
Yes	749 (19.59)	703 (19.33)	46 (23.96)	0.115	717 (19.21)	32 (32.99)	**0.001**
No	3,080 (80.44)	2,934 (80.67)	146 (76.04)		3,015 (80.79)	65 (67.01)	
Medication taken for dyslipidemia (%)							
Yes	217 (5.67)	190 (5.22)	27 (14.06)	**<0.001**	204 (5.47)	13 (13.40)	**0.001**
No	3,612 (94.33)	3,447 (94.78)	165 (85.64)		3,528 (94.53)	84 (86.60)	
Medication taken for CVD (%)							
Yes	342 (8.93)	323 (8.88)	19 (9.90)	0.631	323 (8.65)	19 (19.59)	**<0.001**
No	3,487 (91.07)	3,314 (91.12)	173 (90.10)		3,409 (91.35)	78 (80.41)	
Serum parameters							
FBG (mmol/L)	6.05 ± 1.77	6.00 ± 1.64	7.01 ± 3.23	**<0.001**	6.04 ± 1.73	6.61 ± 2.84	0.081
HbA1c (%)	5.29 ± 0.80	5.27 ± 0.76	5.62 ± 1.34	**0.001**	5.28 ± 0.78	5.61 ± 1.41	**0.036**
TC (mmol/L)	5.03 ± 0.99	5.01 ± 0.97	5.36 ± 1.35	**<0.001**	5.03 ± 0.99	5.13 ± 1.26	0.342
TG (mmol/L)	1.46 ± 1.10	1.42 ± 0.97	2.29 ± 2.36	**<0.001**	1.45 ± 1.10	1.67 ± 0.95	**0.002**
HDL-C (mmol/L)	1.33 ± 0.39	1.33 ± 0.39	1.17 ± 0.39	**<0.001**	1.33 ± 0.39	1.23 ± 0.43	**0.009**
UA (μmol/L)	257.87 ± 70.81	257.40 ± 70.07	266.74 ± 83.27	0.337	256.94 ± 70.26	293.62 ± 82.12	**<0.001**
CRP (mg/L)	2.36 ± 5.82	2.33 ± 5.83	2.90 ± 5.45	**0.014**	2.31 ± 5.72	4.22 ± 8.57	**<0.001**
Creatinine (mg/dL)	0.75 ± 0.16	0.76 ± 0.16	0.70 ± 0.17	**<0.001**	0.75 ± 0.16	0.85 ± 0.20	**<0.001**
Cystatin-C (mg/L)	0.97 ± 0.18	0.98 ± 0.17	0.79 ± 0.22	**<0.001**	0.97 ± 0.18	1.12 ± 0.21	**<0.001**
eGFR baseline	91.43 ± 15.20	90.71 ± 14.66	104.96 ± 18.56	**<0.001**	91.84 ± 14.98	75.71 ± 15.22	**<0.001**
eGFR final	96.14 ± 16.39	97.29 ± 15.44	74.28 ± 18.53	**<0.001**	97.25 ± 15.01	53.09 ± 5.99	**<0.001**
Non-invasive anthropometric indicators							
BMI (kg/m^2^)	23.80 ± 11.81	23.76 ± 12.09	24.41 ± 3.68	**<0.001**	23.79 ± 11.94	24.06 ± 4.00	0.157
WC (cm)	85.37 ± 10.00	85.20 ± 9.98	88.53 ± 10.10	**<0.001**	85.26 ± 9.97	89.34 ± 10.73	**<0.001**
WHtR	0.54 (0.49,0.59)	0.53 (0.49.0.58)	0.57 (0.52,0.61)	**<0.001**	0.54 (0.49,0.59)	0.57 (0.51,0.62)	**<0.001**
ABSI	0.083 (0.080,0.086)	0.083 (0.080,0.086)	0.084 (0.081,0.088)	**0.004**	0.083 (0.080,0.086)	0.085 (0.082,0.088)	**<0.001**
BRI	4.07 (3.24,5.12)	4.04 (3.21,5.07)	4.74 (3.75,5.59)	**<0.001**	4.05 (3.23,5.10)	4.83 (3.62,5.84)	**<0.001**
C-index	1.29 (1.23,1.34)	1.28 (1.23,1.34)	1.31 (1.26,1.37)	**<0.001**	1.28 (1.23,1.34)	1.33 (1.27,1.39)	**<0.001**
RFM	33.38 (25.39,40.76)	33.22 (25.36,40.55)	38.32 (26.67, 42.91)	**0.001**	33.31 (25.37,40.68)	36.81 (26.74,43.04)	**0.013**
WWI	11.14 (10.59,11.73)	11.12 (10.58,11.72)	11.35 (10.85,12.02)	**<0.001**	11.13 (10.59,11.72)	11.58 (10.92,12.19)	**<0.001**
Invasive anthropometric indicators							
VAI	3.42 (2.02,6.06)	3.32 (2.00,5.79)	6.06 (2.98,11.68)	**<0.001**	3.38 (2.01,5.94)	5.31 (2.48,7.36)	**<0.001**
CVAI	95.36 (69.54,125.46)	94.40 (68.76,123.64)	118.56 (87.64,144.32)	**<0.001**	94.97 (69.01,124.58)	119.42 (91.05,152.56)	**<0.001**
LAP	27.28 (14.69,48.22)	26.67 (14.49,46.82)	45.33 (25.34,83.77)	**<0.001**	26.95 (14.61,47.75)	42.30 (20.91,69.80)	**<0.001**
TyG	8.57 (8.22,9.01)	8.56 (8.21,8.99)	9.01 (8.43,9.64)	**<0.001**	8.57 (8.21,9.01)	8.79 (8.30,9.25)	**0.001**
TyG-BMI	199.09 (175.49,229.57)	198.45 (174.89,227.32)	222.11 (187.57,251.28)	**<0.001**	198.81 (175.42,228.97)	216.25 (179.73,252.00)	**0.017**
TyG-WC	731.17 (653.98,817.71)	727.94 (651.80,814.68)	806.75 (711.79,899.95)	**<0.001**	730.12 (653.60,816.86)	792.48 (688.88,902.82)	**<0.001**
TyG-WHtR	4.65 (4.12,5.19)	4.63 (4.11,5.15)	5.13 (4.52,5.73)	**<0.001**	4.64 (4.12,5.17)	5.01 (4.36,5.72)	**<0.001**
TyG-ABSI	0.71 (0.67,0.77)	0.71 (0.67,0.76)	0.76 (0.70,0.82)	**<0.001**	0.71 (0.67,0.76)	0.75 (0.71,0.81)	**<0.001**
TyG-BRI	35.36 (27.17,45.25)	35.06 (27.00,44.80)	42.29 (32.64,53.22)	**<0.001**	35.24 (27.09,45.04)	42.14 (31.43,54.88)	**<0.001**
TyG-RFM	287.71 (216.71,354.68)	285.33 (216.05,352.33)	327.37 (237.08,394.84)	**<0.001**	286.30 (216.32,354.18)	317.51 (240.11,386.67)	**<0.001**
TyG-WWI	91.10 (88.86,103.79)	95.81 (88.71,103.48)	103.25 (94.30,111.65)	**<0.001**	95.97 (88.77,103.64)	102.24 (93.43,112.20)	**<0.001**
TyG-CVAI	825.24 (579.38,1112.50)	815.03 (573.05,1100.13)	1043.34 (746.32,1343.46)	**<0.001**	821.12 (575.37,1107.27)	1032.14 (752.45,1465.84)	**<0.001**

Data were expressed as the mean ± SD or median (upper and lower quartiles) or number (%). RKFD, rapid kidney function decline; CKD, chronic kidney disease; SBP, systolic blood pressure; DBP, diastolic blood pressure; CVD, cardiovascular diseases; FBG, fasting blood glucose; HbA1c, glycosylated hemoglobin; TC, total cholesterol; TG, triglyceride; HDL-C, high-density lipoprotein cholesterol; UA, uric acid; CRP, c reactive protein; eGFR, estimated glomerular filtration rate; BMI, body mass index; WC, waist circumference; WHtR, waist-to-height ratio; ABSI, a body shape index; BRI, body roundness index; C-index, conicity index; RFM, relative fat mass; WWI, weight-adjusted waist index; VAI, visceral adiposity index; CVAI, Chinese visceral adiposity index; LAP, lipid accumulation product; TyG, triglyceride glucose; TyG-BMI, triglyceride glucose × body mass index; TyG-WC, triglyceride glucose × waist circumference; TyG-WHtR, triglyceride glucose × waist-to-height ratio; TyG-ABSI, triglyceride glucose × a body shape index; TyG-BRI, triglyceride glucose × body roundness index; TyG-RFM, triglyceride glucose × relative fat mass; TyG-WWI, triglyceride glucose × weight-adjusted waist index; TyG-CVAI, triglyceride glucose × Chinese visceral adiposity index. Numbers in bold represent *p* < 0.05.

All 20 obesity and lipid indexes in the RKFD group were higher than those in the non-RKFD group, and there were significant differences between the two groups (*p* < 0.05). We also observed that the participants with RKFD are more likely to be older, heavier, have lower education level, unmarried, take medication for diabetes or dyslipidemia, with higher SBP, FBG, HbA1c, TC, TG, CRP, baseline eGFR and with lower HDL-C, creatinine, cystatin-C (*p* < 0.05).

Compared with the non-CKD group, the CKD group showed elevated levels of all 20 obesity and lipid indexes and exhibited significant differences in 19 indexes except BMI (*p* < 0.05). At the same time, there were also differences in baseline characteristics between the two groups. Participants who progressed to CKD were older, had higher proportions of never drinkers and abstainers, a higher proportion of medication use (for kidney disease, diabetes, hypertension, dyslipidemia and CVD), higher SBP, HbA1c, TG, UA, CRP, creatinine, cystatin-C and lower HDL-C (*p* < 0.05).

### Association between obesity and lipid indexes and the risk of RKFD

The association between obesity and lipid indexes and the incidence of RKFD are shown in Table 2. In the crude model, an increased incidence of RKFD was associated with increased SDs of 15 indexes (excluding BMI, ABSI, BRI, TyG-BMI and TyG-BRI). After adjustment for all confounding factors (model 3), for 1 SD increase in WC, VAI, CVAI, LAP, TyG, TyG-WC, TyG-WHtR, TyG-ABSI, TyG-RFM, TyG-WWI and TyG-CVAI, the OR for elevated occurrence of RKFD were 1.02 (95%CI, 1.01 ~ 1.04), 1.02 (95%CI, 1.01 ~ 1.04), 1.01 (95%CI, 1.01 ~ 1.01), 1.01 (95%CI, 1.01 ~ 1.01), 1.61 (95%CI, 1.24 ~ 2.09), 1.01 (95%CI, 1.01 ~ 1.01), 1.26 (95%CI, 1.09 ~ 1.45), 1.03 (95%CI, 1.01 ~ 1.05), 1.01 (95%CI, 1.01 ~ 1.01), 1.02 (95%CI, 1.01 ~ 1.04) and 1.01 (95%CI, 1.01 ~ 1.01) (*p* < 0.05). The occurrence of RKFD and 18 indexes (except BMI and WC) were shown in quartiles. Compared to the first LAP, TyG-WC, TyG-WHtR and TyG-RFM quartile group (Q1), the Q3 and Q4 groups showed the increasing risk of RKFD (*p* < 0.05). While in WHtR, BRI, VAI, CVAI, TyG, TyG-BMI, TyG-ABSI, TyG-BRI, TyG-WWI and TyG-CVAI only Q4 showed the statistical significance (*p* < 0.05). In cubic spline model, we can find that VAI, LAP, TyG, TyG-BMI, TyG-WC, TyG-WHtR, TyG-ABSI, TyG-RFM and TyG-WWI had linear relationship with the risk of progression to RKFD though WHtR, BRI and TyG-BRI had non-linear relationship (Supplementary Figure S2).

**Table 2:** Logistic analysis between obesity and lipid indexes and the incidence of RKFD.

Variable	Crude		Model 1		Model 2		Model 3	
	OR (95% CI)	*p*	OR (95% CI)	*p*	OR (95% CI)	*p*	OR (95% CI)	*p*
BMI								
Per SD	1.00 (1.00 ~ 1.01)	0.487	1.00 (1.00 ~ 1.01)	0.423	1.00 (1.00 ~ 1.01)	0.381	1.01 (0.99 ~ 1.02)	0.393
Thin	Reference	–	Reference	–	Reference	–	Reference	–
Normal	1.51 (0.69 ~ 3.29)	0.304	1.70 (0.77 ~ 3.72)	0.187	1.70 (0.78 ~ 3.74)	0.184	1.36 (0.58 ~ 3.22)	0.481
Overweight	2.13 (0.96 ~ 4.70)	0.062	**2.51 (1.13 ~ 5.59)**	**0.024**	**2.57 (1.15 ~ 5.73)**	**0.021**	1.77 (0.72 ~ 4.34)	0.211
Obesity	**2.41 (1.05 ~ 5.56)**	**0.039**	**2.93 (1.26 ~ 6.82)**	**0.013**	**3.03 (1.29 ~ 7.10)**	**0.011**	1.82 (0.69 ~ 4.82)	0.228
WC								
Per SD	**1.03 (1.02 ~ 1.05)**	**<0.001**	**1.03 (1.02 ~ 1.05)**	**<0.001**	**1.03 (1.02 ~ 1.05)**	**<0.001**	**1.02 (1.01 ~ 1.04)**	**0.044**
Non AO	Reference	–	Reference	–	Reference	–	Reference	–
AO	**1.86 (1.39 ~ 2.50)**	**<0.001**	**1.88 (1.39 ~ 2.55)**	**<0.001**	**1.95 (1.43 ~ 2.66)**	**<0.001**	1.42 (1.00 ~ 2.03)	0.052
WHtR								
Per SD	**6.65 (1.92 ~ 23.04)**	**0.003**	**5.65 (1.67 ~ 19.04)**	**0.005**	**6.01 (1.76 ~ 20.54)**	**0.004**	4.37(0.75 ~ 25.48)	0.101
Q1	Reference	–	Reference	–	Reference	–	Reference	–
Q2	1.42 (0.86 ~ 2.35)	0.168	1.44 (0.87 ~ 2.38)	0.158	1.43 (0.87 ~ 2.38)	0.152	1.13 (0.66 ~ 1.93)	0.653
Q3	**1.90 (1.18 ~ 3.06)**	**0.008**	**1.96 (1.21 ~ 3.19)**	**0.006**	**2.02 (1.24 ~ 3.29)**	**0.005**	1.64 (0.97 ~ 2.76)	0.065
Q4	**3.01 (1.92 ~ 4.71)**	**<0.001**	**3.09 (1.92 ~ 4.96)**	**<0.001**	**3.18 (1.97 ~ 5.14)**	**<0.001**	**1.95 (1.14 ~ 3.34)**	**0.015**
ABSI^#^								
Per SD	1.40 (1.11 ~ 1.76)	0.004	1.25(0.97,1.60)	0.081	1.25(0.98,1.61)	0.076	1.07(0.80,1.42)	0.650
Q1	Reference	–	Reference	–	Reference	–	Reference	–
Q2	**1.76 (1.13 ~ 2.75)**	**0.013**	**1.70 (1.09 ~ 2.66)**	**0.020**	**1.68 (1.08 ~ 2.64)**	**0.023**	1.41 (0.87 ~ 2.27)	0.161
Q3	1.46 (0.92 ~ 2.31)	0.108	1.34 (0.84 ~ 2.13)	0.218	1.34 (0.84 ~ 2.14)	0.221	0.85 (0.51 ~ 1.41)	0.530
Q4	**1.90 (1.22 ~ 2.95)**	**0.004**	1.56 (0.98 ~ 2.48)	0.063	1.57 (0.98 ~ 2.50)	0.061	1.07 (0.64 ~ 1.79)	0.785
BRI								
Per SD	1.01 (0.99 ~ 1.03)	0.187	1.01 (0.99 ~ 1.03)	0.240	1.01 (0.99 ~ 1.03)	0.216	1.02 (0.98 ~ 1.05)	0.354
Q1	Reference	–	Reference	–	Reference	–	Reference	–
Q2	1.43 (0.86 ~ 2.35)	0.166	1.44 (0.87 ~ 2.38)	0.155	1.44 (0.87 ~ 2.38)	0.160	1.13 (0.66 ~ 1.94)	0.653
Q3	**1.90 (1.18 ~ 3.06)**	**0.008**	**1.96 (1.21 ~ 3.19)**	**0.006**	**2.02 (1.24 ~ 3.29)**	**0.005**	1.64 (0.97 ~ 2.76)	0.065
Q4	**3.02 (1.93 ~ 4.72)**	**<0.001**	**3.10 (1.93 ~ 4.98)**	**<0.001**	**3.19 (1.97 ~ 5.15)**	**<0.001**	**1.95 (1.14 ~ 3.34)**	**0.015**
C-index								
Per SD	**23.14(5.13 ~ 104.40)**	**<0.001**	**13.51 (2.70 ~ 67.56)**	**0.002**	**14.40 (2.84 ~ 72.97)**	**0.001**	2.83 (0.45 ~ 18.01)	0.270
Q1	Reference	–	Reference	–	Reference	–	Reference	–
Q2	1.56 (0.96 ~ 2.53)	0.072	1.52 (0.93 ~ 2.46)	0.092	1.52 (0.93 ~ 2.47)	0.094	1.15 (0.69 ~ 1.92)	0.603
Q3	**2.22 (1.40 ~ 3.51)**	**<0.001**	**2.10 (1.32 ~ 3.33)**	**0.002**	**2.13 (1.34 ~ 3.39)**	**0.001**	1.61 (0.99 ~ 2.64)	0.057
Q4	**2.26 (1.43 ~ 3.57)**	**<0.001**	**1.97 (1.22 ~ 3.16)**	**0.005**	**2.01 (1.25 ~ 3.25)**	**0.004**	1.19 (0.70 ~ 2.00)	0.525
RFM								
Per SD	**1.03 (1.01 ~ 1.04)**	**0.003**	**1.07 (1.04 ~ 1.11)**	**<0.001**	**1.08 (1.04 ~ 1.11)**	**<0.001**	1.03 (0.99 ~ 1.08)	0.116
Q1	Reference	–	Reference	–	Reference	–	Reference	–
Q2	1.25 (0.81 ~ 1.93)	0.321	1.36 (0.87 ~ 2.13)	0.172	1.39 (0.89 ~ 2.18)	0.153	1.14 (0.70 ~ 1.86)	0.595
Q3	0.81 (0.50 ~ 1.31)	0.392	1.64 (0.63 ~ 4.26)	0.307	1.71 (0.65 ~ 4.49)	0.278	1.08 (0.37 ~ 3.18)	0.883
Q4	**2.09 (1.40 ~ 3.11)**	**<0.001**	**4.12 (1.60 ~ 10.59)**	**0.003**	**4.30 (1.65 ~ 11.21)**	**0.003**	2.10 (0.71 ~ 6.24)	0.181
WWI								
Per SD	**1.37 (1.18 ~ 1.59)**	**<0.001**	**1.31 (1.10 ~ 1.57)**	**0.003**	**1.31 (1.10 ~ 1.57)**	**0.003**	1.09 (0.89 ~ 1.33)	0.424
Q1	Reference	–	Reference	–	Reference	–	Reference	–
Q2	1.16 (0.72 ~ 1.86)	0.543	1.13 (0.70 ~ 1.82)	0.613	1.13 (0.70 ~ 1.82)	0.626	0.91 (0.55 ~ 1.51)	0.711
Q3	**1.81 (1.17 ~ 2.80)**	**0.008**	**1.72 (1.10 ~ 2.70)**	**0.018**	**1.74 (1.11 ~ 2.75)**	**0.016**	1.31 (0.80 ~ 2.13)	0.284
Q4	**1.97 (1.28 ~ 3.04)**	**0.002**	**1.76 (1.09 ~ 2.84)**	**0.021**	**1.77 (1.09 ~ 2.86)**	**0.021**	1.09 (0.63 ~ 1.86)	0.761
VAI								
Per SD	**1.05 (1.03 ~ 1.06)**	**<0.001**	**1.05 (1.03 ~ 1.06)**	**<0.001**	**1.05 (1.04 ~ 1.06)**	**<0.001**	**1.02 (1.01 ~ 1.04)**	**<0.001**
Q1	Reference	–	Reference	–	Reference	–	Reference	–
Q2	1.20 (0.71 ~ 2.04)	0.499	1.26 (0.74 ~ 2.16)	0.390	1.27 (0.74 ~ 2.17)	0.379	1.35 (0.77 ~ 2.35)	0.292
Q3	1.52 (0.92 ~ 2.52)	0.101	1.68 (1.00 ~ 2.81)	0.051	**1.70 (1.01 ~ 2.86)**	**0.044**	1.47 (0.85 ~ 2.55)	0.165
Q4	**3.99 (2.56 ~ 6.22)**	**<0.001**	**4.49 (2.82 ~ 7.17)**	**<0.001**	**4.63 (2.89 ~ 7.41)**	**<0.001**	**2.44 (1.44 ~ 4.14)**	**<0.001**
CVAI								
Per SD	**1.01 (1.01 ~ 1.01)**	**0.010**	**1.01 (1.01 ~ 1.01)**	**0.037**	**1.01 (1.01 ~ 1.01)**	**0.031**	**1.01 (1.01 ~ 1.01)**	**0.038**
Q1	Reference	–	Reference	–	Reference	–	Reference	–
Q2	1.32 (0.79 ~ 2.22)	0.295	1.27 (0.75 ~ 2.15)	0.366	1.30 (0.77 ~ 2.20)	0.325	1.12 (0.65 ~ 1.94)	0.682
Q3	**1.89 (1.16 ~ 3.07)**	**0.010**	**1.80 (1.10 ~ 2.96)**	**0.019**	**1.88 (1.14 ~ 3.10)**	**0.013**	1.47 (0.86 ~ 2.50)	0.158
Q4	**3.45 (2.20 ~ 5.40)**	**<0.001**	**3.17 (2.00 ~ 5.03)**	**<0.001**	**3.39 (2.12 ~ 5.43)**	**<0.001**	**1.86 (1.09 ~ 3.17)**	**0.023**
LAP								
Per SD	**1.01 (1.01 ~ 1.01)**	**<0.001**	**1.01 (1.01 ~ 1.01)**	**<0.001**	**1.01 (1.01 ~ 1.01)**	**<0.001**	**1.01 (1.01 ~ 1.01)**	**0.001**
Q1	Reference	–	Reference	–	Reference	–	Reference	–
Q2	1.32 (0.79 ~ 2.23)	0.288	1.40 (0.83 ~ 2.36)	0.211	1.43 (0.84 ~ 2.42)	0.187	1.33 (0.76 ~ 2.31)	0.313
Q3	**1.77 (1.08 ~ 2.89)**	**0.023**	**1.91 (1.15 ~ 3.16)**	**0.012**	**1.99 (1.20 ~ 3.31)**	**0.008**	**1.78 (1.04 ~ 3.06)**	**0.037**
Q4	**3.59 (2.29 ~ 5.61)**	**<0.001**	**3.98 (2.48 ~ 6.36)**	**<0.001**	**4.19 (2.60 ~ 6.76)**	**<0.001**	**2.09 (1.21 ~ 3.61)**	**0.008**
TyG								
Per SD	**2.47 (2.04 ~ 2.98)**	**<0.001**	**2.52 (2.08 ~ 3.06)**	**<0.001**	**2.60 (2.13 ~ 3.16)**	**<0.001**	**1.61 (1.24 ~ 2.09)**	**<0.001**
Q1	Reference	–	Reference	–	Reference	–	Reference	–
Q2	1.50 (0.89 ~ 2.51)	0.125	1.50 (0.89 ~ 2.51)	0.124	1.52 (0.90 ~ 2.54)	0.115	1.66 (0.96 ~ 2.86)	0.068
Q3	1.37 (0.81 ~ 2.32)	0.236	1.37 (0.81 ~ 2.33)	0.238	1.39 (0.82 ~ 2.35)	0.225	1.30 (0.74 ~ 2.29)	0.360
Q4	**4.16 (2.65 ~ 6.52)**	**<0.001**	**4.19 (2.66 ~ 6.59)**	**<0.001**	**4.26 (2.70 ~ 6.72)**	**<0.001**	**2.19 (1.28 ~ 3.75)**	**0.004**
TyG-BMI								
Per SD	1.00 (1.00 ~ 1.00)	0.079	1.00 (1.00 ~ 1.00)	0.067	1.00 (1.00 ~ 1.00)	0.055	1.00 (1.00 ~ 1.00)	0.121
Q1	Reference	–	Reference	–	Reference	–	Reference	–
Q2	1.48 (0.89 ~ 2.46)	0.129	1.56 (0.94 ~ 2.59)	0.088	1.59 (0.95 ~ 2.64)	0.077	1.52 (0.89 ~ 2.61)	0.125
Q3	**1.77 (1.08 ~ 2.89)**	**0.023**	**1.94 (1.18 ~ 3.18)**	**0.009**	**2.02 (1.22 ~ 3.33)**	**0.006**	1.61 (0.93 ~ 2.77)	0.089
Q4	**3.40 (2.17 ~ 5.33)**	**<0.001**	**3.89 (2.45 ~ 6.17)**	**<0.001**	**4.10 (2.56 ~ 6.58)**	**<0.001**	**2.52 (1.45 ~ 4.39)**	**0.001**
TyG-WC								
Per SD	**1.01 (1.01 ~ 1.01)**	**<0.001**	**1.01 (1.01 ~ 1.01)**	**<0.001**	**1.01 (1.01 ~ 1.01)**	**<0.001**	**1.01 (1.01 ~ 1.01)**	**0.001**
Q1	Reference	–	Reference	–	Reference	–	Reference	–
Q2	1.21 (0.71 ~ 2.07)	0.490	1.21 (0.70 ~ 2.07)	0.491	1.22 (0.71 ~ 2.10)	0.469	1.20 (0.68 ~ 2.11)	0.535
Q3	**1.88 (1.15 ~ 3.09)**	**0.012**	**1.91 (1.16 ~ 3.13)**	**0.011**	**1.99 (1.20 ~ 3.27)**	**0.007**	**1.78 (1.04 ~ 3.04)**	**0.036**
Q4	**3.92 (2.49 ~ 6.16)**	**<0.001**	**3.97 (2.52 ~ 6.27)**	**<0.001**	**4.23 (2.66 ~ 6.71)**	**<0.001**	**2.60 (1.52 ~ 4.47)**	**<0.001**
TyG-WHtR								
Per SD	**1.54 (1.30 ~ 1.82)**	**<0.001**	**1.53 (1.29 ~ 1.83)**	**<0.001**	**1.56 (1.30 ~ 1.87)**	**<0.001**	**1.26 (1.09 ~ 1.45)**	**0.002**
Q1	Reference	–	Reference	–	Reference	–	Reference	–
Q2	1.57 (0.91 ~ 2.70)	0.105	1.62 (0.94 ~ 2.79)	0.084	1.64 (0.95 ~ 2.83)	0.077	1.42 (0.80 ~ 2.51)	0.234
Q3	**2.15 (1.28 ~ 3.60)**	**0.004**	**2.26 (1.34 ~ 3.81)**	**0.002**	**2.34 (1.38 ~ 3.96)**	**0.002**	**1.95 (1.11 ~ 3.43)**	**0.021**
Q4	**4.42 (2.75 ~ 7.10)**	**<0.001**	**4.74 (2.89 ~ 7.77)**	**<0.001**	**4.97 (3.01 ~ 8.20)**	**<0.001**	**2.65 (1.49 ~ 4.68)**	**<0.001**
TyG-ABSI^*^								
Per SD	**1.07 (1.06 ~ 1.09)**	**<0.001**	**1.07 (1.05 ~ 1.09)**	**<0.001**	**1.07 (1.05 ~ 1.09)**	**<0.001**	**1.03 (1.01 ~ 1.05)**	**0.006**
Q1	Reference	–	Reference	–	Reference	–	Reference	–
Q2	1.28 (0.76 ~ 2.16)	0.356	1.25 (0.74 ~ 2.11)	0.408	1.25 (0.74 ~ 2.11)	0.408	1.01 (0.59 ~ 1.75)	0.962
Q3	1.48 (0.89 ~ 2.46)	0.129	1.43 (0.86 ~ 2.39)	0.172	1.43 (0.86 ~ 2.39)	0.172	1.15 (0.67 ~ 1.97)	0.604
Q4	**3.94 (2.53 ~ 6.14)**	**<0.001**	**3.83 (2.42 ~ 6.06)**	**<0.001**	**3.83 (2.42 ~ 6.06)**	**<0.001**	**1.84 (1.10 ~ 3.08)**	**0.021**
TyG-BRI								
Per SD	1.00 (1.00 ~ 1.00)	0.064	1.00 (1.00 ~ 1.00)	0.068	1.00 (1.00 ~ 1.00)	0.068	1.00 (1.00 ~ 1.01)	0.177
Q1	Reference	–	Reference	–	Reference	–	Reference	–
Q2	1.40 (0.84 ~ 2.34)	0.199	1.43 (0.85 ~ 2.39)	0.176	1.43 (0.85 ~ 2.39)	0.176	1.12 (0.65 ~ 1.93)	0.683
Q3	**1.81 (1.11 ~ 2.95)**	**0.018**	**1.95 (1.18 ~ 3.22)**	**0.009**	**1.95 (1.18 ~ 3.22)**	**0.009**	1.51 (0.88 ~ 2.58)	0.132
Q4	**3.44 (2.20 ~ 5.39)**	**<0.001**	**3.77 (2.33 ~ 6.09)**	**<0.001**	**3.77 (2.33 ~ 6.09)**	**<0.001**	**2.09 (1.22 ~ 3.59)**	**0.008**
TyG-RFM								
Per SD	**1.01 (1.01 ~ 1.01)**	**<0.001**	**1.01 (1.01 ~ 1.01)**	**<0.001**	**1.01 (1.01 ~ 1.01)**	**<0.001**	**1.01 (1.01 ~ 1.01)**	**0.006**
Q1	Reference	–	Reference	–	Reference	–	Reference	–
Q2	1.29 (0.81 ~ 2.07)	0.284	**1.66 (1.03 ~ 2.69)**	**0.038**	**1.66 (1.03 ~ 2.69)**	**0.038**	1.26 (0.75 ~ 2.12)	0.39
Q3	1.29 (0.81 ~ 2.07)	0.284	**4.33 (2.25 ~ 8.33)**	**<0.001**	**4.33 (2.25 ~ 8.33)**	**<0.001**	**2.42 (1.19 ~ 4.94)**	**0.015**
Q4	**2.56 (1.68 ~ 3.91)**	**<0.001**	**10.71 (5.12 ~ 22.41)**	**<0.001**	**10.71(5.12 ~ 22.41)**	**<0.001**	**3.98 (1.71 ~ 9.22)**	**0.001**
TyG-WWI								
Per SD	**1.05 (1.04 ~ 1.06)**	**<0.001**	**1.05 (1.04 ~ 1.06)**	**<0.001**	**1.05 (1.04 ~ 1.07)**	**<0.001**	**1.02 (1.01 ~ 1.04)**	**0.004**
Q1	Reference	–	Reference	–	Reference	–	Reference	–
Q2	1.54 (0.91 ~ 2.63)	0.110	1.56 (0.91 ~ 2.66)	0.106	1.58 (0.92 ~ 2.70)	0.097	1.23 (0.71 ~ 2.16)	0.460
Q3	**1.82 (1.08 ~ 3.05)**	**0.024**	**1.87 (1.10 ~ 3.16)**	**0.020**	**1.91 (1.13 ~ 3.24)**	**0.016**	1.48 (0.85 ~ 2.58)	0.164
Q4	**4.38 (2.75 ~ 6.97)**	**<0.001**	**4.57 (2.79 ~ 7.47)**	**<0.001**	**4.71 (2.87 ~ 7.75)**	**<0.001**	**2.07 (1.18 ~ 3.61)**	**0.011**
TyG-CVAI								
Per SD	**1.01 (1.01 ~ 1.01)**	**<0.001**	**1.01 (1.01 ~ 1.01)**	**<0.001**	**1.01 (1.01 ~ 1.01)**	**<0.001**	**1.01 (1.01 ~ 1.01)**	**0.013**
Q1	Reference	–	Reference	–	Reference	–	Reference	–
Q2	1.12 (0.65 ~ 1.91)	0.682	1.10 (0.64 ~ 1.90)	0.721	1.10 (0.64 ~ 1.90)	0.721	0.96 (0.55 ~ 1.70)	0.899
Q3	**1.93 (1.19 ~ 3.14)**	**0.008**	**1.94 (1.18 ~ 3.18)**	**0.009**	**1.94 (1.18 ~ 3.18)**	**0.009**	1.57 (0.92 ~ 2.67)	0.095
Q4	**3.62 (2.32 ~ 5.66)**	**<0.001**	**3.62 (2.27 ~ 5.76)**	**<0.001**	**3.62 (2.27 ~ 5.76)**	**<0.001**	**2.07 (1.21 ~ 3.52)**	**0.008**

OR, Odds ratio; CI, Confidence interval. Crude: unadjusted. Model 1: adjusted for gender, age. Model 2: adjusted for gender, age, education, residence, marital status, drinking status, and smoking status. Model 3: adjusted for gender, age, education, residence, marital status, drinking status, smoking status, SBP, DBP, TC, HbA1c, UA, CRP, baseline eGFR and medication usage (for kidney disease, diabetes, hypertension, dyslipidemia, CVD). # Analyzed by 100ABSI. * Analyzed by 100TyG-ABSI. Numbers in bold represent *p* < 0.05.

### Association between obesity and lipid indexes and the risk of CKD

Logistic regression analyses revealed significant positive associations between WC, WHtR, ABSI, C-index, RFM, WWI, VAI, CVAI, LAP, TyG, TyG-WC, TyG-WHtR, TyG-ABSI, TyG-RFM, TyG-WWI, TyG-CVAI and CKD prevalence in the crude model (Table 3). Following adjustment for all confounding factors (model 3), each SD increase in the WC was associated with a 2% higher risk of CKD (OR: 1.02; 95% CI: 1.01, 1.05), a 1% higher risk in LAP (OR: 1.01; 95% CI: 1.01, 1.01), a 1% higher risk in TyG-WC (OR: 1.01; 95% CI: 1.01, 1.01), a 3% higher risk in TyG-ABSI (OR: 1.03; 95% CI: 1.01, 1.06) (*p* < 0.05). The occurrence of the progression to CKD and 18 indexes (except BMI and WC) were shown in quartiles. Compared to the first VAI, LAP, TyG-BMI, TyG-WHtR, TyG-WWI quartile group (Q1), the Q4 group showed the increasing risk of the progression to CKD (*p* < 0.05). Meanwhile, the OR of WC in AO was 1.72 (95% CI: 1.04 ~ 2.83) (*p* < 0.05). According to the cubic spline model, we can find that LAP had linear relationship with the risk of CKD while VAI had non-linear relationship (Supplementary Figure S3).

**Table 3:** Logistic analysis between obesity and lipid indexes and the incidence of CKD.

Variable	Crude		Model 1		Model 2		Model 3	
	OR (95% CI)	*p* value	OR (95% CI)	*p* value	OR (95% CI)	*p* value	OR (95% CI)	*p* value
BMI								
Per SD	1.00 (0.99 ~ 1.01)	0.829	1.00 (0.99 ~ 1.01)	0.492	1.00 (0.99 ~ 1.01)	0.492	1.00 (0.99 ~ 1.02)	0.821
Thin	Reference		Reference		Reference		Reference	
Normal	0.86 (0.36 ~ 2.05)	0.738	1.28 (0.53 ~ 3.07)	0.584	1.30 (0.54 ~ 3.15)	0.56	1.07 (0.43 ~ 2.62)	0.889
Overweight	1.21 (0.50 ~ 2.92)	0.674	2.30 (0.93 ~ 5.67)	0.071	2.30 (0.92 ~ 5.75)	0.074	1.40 (0.54 ~ 3.67)	0.488
Obesity	1.23 (0.47 ~ 3.23)	0.679	**2.75 (1.01 ~ 7.49)**	**0.048**	2.69 (0.98 ~ 7.42)	0.056	1.28 (0.43 ~ 3.80)	0.657
WC								
Per SD	**1.04 (1.02 ~ 1.06)**	**<0.001**	**1.04 (1.02 ~ 1.06)**	**<0.001**	**1.04 (1.02 ~ 1.06)**	**<0.001**	**1.02 (1.01 ~ 1.05)**	**0.035**
Non-AO	Reference		Reference		Reference		Reference	
AO	**2.08 (1.38 ~ 3.15)**	**<0.001**	**2.36 (1.51 ~ 3.68)**	**<0.001**	**2.35 (1.49 ~ 3.71)**	**<0.001**	**1.72 (1.04 ~ 2.83)**	**0.034**
WHtR								
Per SD	**5.92 (1.66 ~ 21.14)**	**0.006**	**4.88 (1.25 ~ 19.08)**	**0.023**	**4.88 (1.25 ~ 19.08)**	**0.023**	3.03 (0.42 ~ 22.01)	0.273
Q1	Reference		Reference		Reference		Reference	
Q2	0.94 (0.47 ~ 1.87)	0.858	0.93 (0.46 ~ 1.86)	0.832	1.00 (0.49 ~ 2.02)	0.997	1.03 (0.50 ~ 2.11)	0.936
Q3	1.30 (0.69 ~ 2.47)	0.418	1.40 (0.72 ~ 2.70)	0.317	1.44 (0.74 ~ 2.81)	0.285	1.21 (0.60 ~ 2.42)	0.595
Q4	**2.54 (1.43 ~ 4.49)**	**0.001**	**2.42 (1.29 ~ 4.53)**	**0.006**	**2.46 (1.30 ~ 4.66)**	**0.006**	1.69 (0.84 ~ 3.39)	0.142
ABSI^#^								
Per SD	**2.01 (1.52 ~ 2.67)**	**<0.001**	1.25 (0.91 ~ 1.73)	0.168	1.25 (0.90 ~ 1.73)	0.181	1.24 (0.88 ~ 1.75)	0.223
Q1	Reference		Reference		Reference		Reference	
Q2	1.94 (0.96 ~ 3.92)	0.065	1.62 (0.80 ~ 3.31)	0.182	1.68 (0.82 ~ 3.43)	0.158	1.69 (0.81 ~ 3.54)	0.162
Q3	1.68 (0.82 ~ 3.45)	0.159	1.15 (0.55 ~ 2.40)	0.702	1.11 (0.53 ~ 2.31)	0.788	1.09 (0.51 ~ 2.31)	0.826
Q4	**3.61 (1.89 ~ 6.91)**	**<0.001**	1.58 (0.80 ~ 3.14)	0.187	1.52 (0.76 ~ 3.02)	0.234	1.39 (0.69 ~ 2.83)	0.357
BRI								
Per SD	1.01 (0.99 ~ 1.04)	0.245	1.01 (0.99 ~ 1.04)	0.349	1.01 (0.99 ~ 1.04)	0.349	1.01 (0.96 ~ 1.06)	0.675
Q1	Reference		Reference		Reference		Reference	
Q2	0.83 (0.42 ~ 1.66)	0.601	0.82 (0.41 ~ 1.65)	0.575	0.88 (0.43 ~ 1.78)	0.723	0.91 (0.44 ~ 1.87)	0.801
Q3	1.23 (0.65 ~ 2.31)	0.521	1.31 (0.69 ~ 2.51)	0.408	1.35 (0.70 ~ 2.62)	0.369	1.14 (0.57 ~ 2.25)	0.716
Q4	**2.40 (1.37 ~ 4.19)**	**0.002**	**2.28 (1.23 ~ 4.22)**	**0.009**	**2.32 (1.24 ~ 4.34)**	**0.009**	1.59 (0.80 ~ 3.16)	0.187
C-index								
Per SD	**199.91 (28.46 ~ 1404.47)**	**<0.001**	**14.14 (1.64 ~ 121.59)**	**0.016**	**14.14 (1.64 ~ 121.59)**	**0.016**	6.62 (0.66 ~ 66.92)	0.109
Q1	Reference		Reference		Reference		Reference	
Q2	0.85 (0.39 ~ 1.86)	0.691	0.74 (0.34 ~ 1.62)	0.446	0.74 (0.34 ~ 1.63)	0.46	0.80 (0.36 ~ 1.79)	0.593
Q3	**2.33 (1.24 ~ 4.40)**	**0.009**	1.87 (0.98 ~ 3.56)	0.057	1.80 (0.94 ~ 3.45)	0.077	1.65 (0.84 ~ 3.25)	0.144
Q4	**2.86 (1.54 ~ 5.31)**	**<0.001**	1.59 (0.83 ~ 3.04)	0.16	1.52 (0.79 ~ 2.94)	0.209	1.20 (0.60 ~ 2.39)	0.598
RFM								
Per SD	**1.03 (1.01 ~ 1.05)**	**0.023**	**1.08 (1.03 ~ 1.13)**	**0.002**	**1.08 (1.03 ~ 1.13)**	**0.002**	1.04 (0.99 ~ 1.10)	0.142
Q1	Reference		Reference		Reference		Reference	
Q2	1.15 (0.63 ~ 2.11)	0.646	1.31 (0.71 ~ 2.43)	0.392	1.34 (0.71 ~ 2.51)	0.364	1.02 (0.52 ~ 1.97)	0.963
Q3	0.80 (0.41 ~ 1.55)	0.502	3.01 (0.73 ~ 12.42)	0.129	2.93 (0.71 ~ 12.20)	0.139	1.64 (0.41 ~ 6.57)	0.482
Q4	**1.94 (1.12 ~ 3.35)**	**0.018**	**6.04 (1.42 ~ 25.70)**	**0.015**	**5.69 (1.33 ~ 24.40)**	**0.019**	2.47 (0.58 ~ 10.58)	0.224
WWI								
Per SD	**1.65 (1.36 ~ 2.00)**	**<0.001**	1.23 (0.97 ~ 1.55)	0.087	1.23 (0.97 ~ 1.55)	0.087	1.14 (0.88 ~ 1.46)	0.317
Q1	Reference		Reference		Reference		Reference	
Q2	0.81 (0.39 ~ 1.69)	0.577	0.69 (0.33 ~ 1.46)	0.335	0.69 (0.33 ~ 1.46)	0.332	0.67 (0.31 ~ 1.45)	0.304
Q3	1.84 (0.99 ~ 3.41)	0.052	1.42 (0.75 ~ 2.67)	0.284	1.36 (0.71 ~ 2.58)	0.353	1.27 (0.65 ~ 2.47)	0.48
Q4	**2.50 (1.39 ~ 4.50)**	**0.002**	1.31 (0.68 ~ 2.53)	0.426	1.24 (0.64 ~ 2.41)	0.531	0.98 (0.48 ~ 1.96)	0.946
VAI								
Per SD	**1.02 (1.01 ~ 1.03)**	**0.037**	1.02 (1.00 ~ 1.03)	0.05	1.02 (1.00 ~ 1.03)	0.05	1.01 (1.00 ~ 1.03)	0.142
Q1	Reference		Reference		Reference		Reference	
Q2	1.00 (0.51 ~ 1.97)	0.998	1.09 (0.54 ~ 2.18)	0.816	1.09 (0.54 ~ 2.20)	0.804	0.85 (0.42 ~ 1.75)	0.665
Q3	1.30 (0.69 ~ 2.47)	0.416	1.53 (0.78 ~ 3.01)	0.213	1.53 (0.78 ~ 3.02)	0.22	1.23 (0.61 ~ 2.49)	0.558
Q4	**2.47 (1.40 ~ 4.39)**	**0.002**	**3.11 (1.65 ~ 5.84)**	**<0.001**	**3.14 (1.65 ~ 5.99)**	**<0.001**	**2.27 (1.12 ~ 4.58)**	**0.023**
CVAI								
Per SD	**1.01 (1.01 ~ 1.01)**	**0.035**	1.00 (1.00 ~ 1.00)	0.059	1.00 (1.00 ~ 1.00)	0.059	1.00 (1.00 ~ 1.00)	0.237
Q1	Reference		Reference		Reference		Reference	
Q2	2.02 (0.94 ~ 4.34)	0.071	1.76 (0.81 ~ 3.82)	0.155	1.79 (0.82 ~ 3.91)	0.144	1.73 (0.78 ~ 3.83)	0.177
Q3	2.12 (0.99 ~ 4.53)	0.052	1.78 (0.81 ~ 3.88)	0.149	1.83 (0.83 ~ 4.04)	0.135	1.66 (0.74 ~ 3.75)	0.219
Q4	**4.78 (2.40 ~ 9.53)**	**<0.001**	**3.20 (1.56 ~ 6.58)**	**0.002**	**3.27 (1.57 ~ 6.82)**	**0.002**	2.08 (0.94 ~ 4.60)	0.07
LAP								
Per SD	**1.01 (1.01 ~ 1.01)**	**0.004**	**1.01 (1.01 ~ 1.01)**	**0.002**	**1.01 (1.01 ~ 1.01)**	**0.002**	**1.01 (1.01 ~ 1.01)**	**0.040**
Q1	Reference		Reference		Reference		Reference	
Q2	0.82 (0.40 ~ 1.68)	0.596	0.93 (0.45 ~ 1.93)	0.842	1.03 (0.49 ~ 2.16)	0.942	0.88 (0.42 ~ 1.87)	0.744
Q3	1.67 (0.91 ~ 3.07)	0.1	**2.13 (1.12 ~ 4.06)**	**0.021**	**2.33 (1.20 ~ 4.51)**	**0.012**	1.94 (0.97 ~ 3.89)	0.06
Q4	**2.29 (1.28 ~ 4.09)**	**0.005**	**3.10 (1.64 ~ 5.86)**	**<0.001**	**3.37 (1.74 ~ 6.52)**	**<0.001**	**2.30 (1.10 ~ 4.82)**	**0.027**
TyG								
Per SD	**1.60 (1.21 ~ 2.10)**	**<0.001**	**1.76 (1.30 ~ 2.37)**	**<0.001**	**1.76 (1.30 ~ 2.37)**	**<0.001**	1.46 (0.99 ~ 2.17)	0.056
Q1	Reference		Reference		Reference		Reference	
Q2	0.69 (0.35 ~ 1.38)	0.3	0.72 (0.36 ~ 1.45)	0.36	0.71 (0.35 ~ 1.44)	0.348	0.68 (0.33 ~ 1.40)	0.294
Q3	1.36 (0.76 ~ 2.44)	0.303	1.44 (0.79 ~ 2.62)	0.235	1.52 (0.83 ~ 2.78)	0.179	1.26 (0.67 ~ 2.39)	0.477
Q4	**1.83 (1.05 ~ 3.19)**	**0.032**	**2.01 (1.13 ~ 3.57)**	**0.018**	**2.07 (1.15 ~ 3.72)**	**0.015**	1.44 (0.74 ~ 2.81)	0.279
TyG-BMI								
Per SD	1.00 (1.00 ~ 1.00)	0.486	1.00 (1.00 ~ 1.00)	0.256	1.00 (1.00 ~ 1.00)	0.256	1.00 (1.00 ~ 1.00)	0.602
Q1	Reference		Reference		Reference		Reference	
Q2	0.85 (0.45 ~ 1.61)	0.628	1.01 (0.53 ~ 1.92)	0.988	1.02 (0.53 ~ 1.95)	0.964	0.97 (0.50 ~ 1.89)	0.933
Q3	0.95 (0.51 ~ 1.77)	0.875	1.31 (0.69 ~ 2.48)	0.41	1.38 (0.72 ~ 2.65)	0.332	1.11 (0.56 ~ 2.18)	0.771
Q4	**1.84 (1.07 ~ 3.16)**	**0.027**	**3.10 (1.74 ~ 5.55)**	**<0.001**	**3.20 (1.75 ~ 5.83)**	**<0.001**	**2.05 (1.03 ~ 4.06)**	**0.04**
TyG-WC								
Per SD	**1.01 (1.01 ~ 1.01)**	**<0.001**	**1.01 (1.01 ~ 1.01)**	**<0.001**	**1.01 (1.01 ~ 1.01)**	**<0.001**	**1.01 (1.01 ~ 1.01)**	**0.008**
Q1	Reference		Reference		Reference		Reference	
Q2	0.83 (0.42 ~ 1.66)	0.603	0.82 (0.41 ~ 1.66)	0.59	0.83 (0.41 ~ 1.69)	0.614	0.79 (0.39 ~ 1.63)	0.529
Q3	1.34 (0.72 ~ 2.49)	0.349	1.47 (0.78 ~ 2.77)	0.228	1.52 (0.80 ~ 2.89)	0.199	1.29 (0.66 ~ 2.54)	0.453
Q4	**2.28 (1.30 ~ 4.00)**	**0.004**	**2.59 (1.44 ~ 4.65)**	**0.001**	**2.67 (1.46 ~ 4.87)**	**0.001**	1.75 (0.88 ~ 3.49)	0.111
TyG-WHtR								
Per SD	**1.27 (1.10 ~ 1.46)**	**<0.001**	**1.25 (1.09 ~ 1.44)**	**0.001**	**1.25 (1.09 ~ 1.44)**	**0.001**	1.18 (0.98 ~ 1.41)	0.076
Q1	Reference		Reference		Reference		Reference	
Q2	1.07 (0.54 ~ 2.12)	0.856	1.12 (0.56 ~ 2.26)	0.744	1.17 (0.58 ~ 2.37)	0.659	1.23 (0.60 ~ 2.52)	0.576
Q3	1.26 (0.65 ~ 2.44)	0.502	1.37 (0.69 ~ 2.72)	0.363	1.45 (0.72 ~ 2.90)	0.298	1.23 (0.59 ~ 2.53)	0.58
Q4	**2.84 (1.59 ~ 5.06)**	**<0.001**	**3.14 (1.67 ~ 5.89)**	**<0.001**	**3.27 (1.71 ~ 6.25)**	**<0.001**	**2.20 (1.07 ~ 4.55)**	**0.033**
TyG-ABSI^*^								
Per SD	**1.06 (1.04 ~ 1.09)**	**<0.001**	**1.04 (1.02 ~ 1.07)**	**<0.001**	**1.04 (1.02 ~ 1.07)**	**<0.001**	**1.03 (1.01 ~ 1.06)**	**0.038**
Q1	Reference		Reference		Reference		Reference	
Q2	1.65 (0.77 ~ 3.51)	0.195	1.38 (0.64 ~ 2.96)	0.416	1.38 (0.64 ~ 2.96)	0.416	1.42 (0.65 ~ 3.09)	0.383
Q3	**2.31 (1.13 ~ 4.72)**	**0.022**	1.62 (0.78 ~ 3.36)	0.199	1.62 (0.78 ~ 3.36)	0.199	1.45 (0.68 ~ 3.06)	0.336
Q4	**4.04 (2.07 ~ 7.89)**	**<0.001**	**2.67 (1.32 ~ 5.41)**	**0.006**	**2.67 (1.32 ~ 5.41)**	**0.006**	2.06 (0.97 ~ 4.38)	0.061
TyG-BRI								
Per SD	1.00 (1.00 ~ 1.00)	0.165	1.00 (1.00 ~ 1.00)	0.219	1.00 (1.00 ~ 1.00)	0.219	1.00 (1.00 ~ 1.01)	0.55
Q1	Reference		Reference		Reference		Reference	
Q2	1.06 (0.53 ~ 2.12)	0.861	1.12 (0.55 ~ 2.26)	0.761	1.12 (0.55 ~ 2.26)	0.761	1.17 (0.57 ~ 2.40)	0.663
Q3	1.32 (0.68 ~ 2.54)	0.408	1.50 (0.75 ~ 2.98)	0.253	1.50 (0.75 ~ 2.98)	0.253	1.25 (0.61 ~ 2.56)	0.547
Q4	**2.76 (1.55 ~ 4.94)**	**<0.001**	**2.93 (1.53 ~ 5.63)**	**0.001**	**2.93 (1.53 ~ 5.63)**	**0.001**	1.95 (0.95 ~ 4.01)	0.069
TyG-RFM								
Per SD	**1.01 (1.01 ~ 1.01)**	**0.003**	**1.01 (1.01 ~ 1.01)**	**<0.001**	**1.01 (1.01 ~ 1.01)**	**<0.001**	1.00 (1.00 ~ 1.01)	0.056
Q1	Reference		Reference		Reference		Reference	Reference
Q2	1.30 (0.69 ~ 2.47)	0.42	**1.85 (0.95 ~ 3.60)**	**0.07**	**1.85 (0.95 ~ 3.60)**	**0.07**	1.38 (0.69 ~ 2.79)	0.364
Q3	1.36 (0.72 ~ 2.57)	0.339	**4.82 (1.92 ~ 12.08)**	**<0.001**	**4.82 (1.92 ~ 12.08)**	**<0.001**	**2.86 (1.09 ~ 7.50)**	**0.033**
Q4	**2.10 (1.17 ~ 3.77)**	**0.013**	**6.79 (2.42 ~ 19.03)**	**<0.001**	**6.79 (2.42 ~ 19.03)**	**<0.001**	2.92 (0.93 ~ 9.19)	0.067
TyG-WWI								
Per SD	**1.04 (1.03 ~ 1.06)**	**<0.001**	**1.03 (1.01 ~ 1.05)**	**<0.001**	**1.03 (1.01 ~ 1.05)**	**<0.001**	1.02 (1.00 ~ 1.04)	0.063
Q1	Reference		Reference		Reference		Reference	
Q2	1.68 (0.82 ~ 3.47)	0.157	1.50 (0.72 ~ 3.12)	0.275	1.51 (0.72 ~ 3.15)	0.276	1.59 (0.75 ~ 3.39)	0.225
Q3	1.68 (0.82 ~ 3.46)	0.158	1.45 (0.69 ~ 3.04)	0.328	1.49 (0.70 ~ 3.14)	0.298	1.30 (0.60 ~ 2.81)	0.504
Q4	**3.89 (2.04 ~ 7.40)**	**<0.001**	**3.00 (1.48 ~ 6.05)**	**0.002**	**3.04 (1.49 ~ 6.21)**	**0.002**	**2.22 (1.01 ~ 4.87)**	**0.046**
TyG-CVAI								
Per SD	**1.01 (1.01 ~ 1.01)**	**0.039**	**1.01 (1.01 ~ 1.01)**	**0.038**	**1.01 (1.01 ~ 1.01)**	**0.038**	1.00 (1.00 ~ 1.00)	0.163
Q1	Reference		Reference		Reference		Reference	Reference
Q2	2.02 (0.94 ~ 4.34)	0.071	1.74 (0.79 ~ 3.79)	0.167	1.74 (0.79 ~ 3.79)	0.167	1.72 (0.78 ~ 3.82)	0.181
Q3	**2.33 (1.10 ~ 4.93)**	**0.026**	2.03 (0.93 ~ 4.43)	0.075	2.03 (0.93 ~ 4.43)	0.075	1.80 (0.81 ~ 4.00)	0.152
Q4	**4.56 (2.28 ~ 9.11)**	**<0.001**	**3.28 (1.57 ~ 6.85)**	**0.002**	**3.28 (1.57 ~ 6.85)**	**0.002**	2.06 (0.92 ~ 4.61)	0.078

OR: Odds Ratio, CI: Confidence Interval. Crude: unadjusted. Model 1: adjusted for gender, age. Model 2: adjusted for gender, age, education, residence, marital status, drinking status, smoking status. Model 3: adjusted for gender, age, education, residence, marital status, drinking status, smoking status, SBP, DBP, CHO, HbA1c, UA, CRP, baseline eGFR and medication usage (for kidney disease, diabetes, hypertension, dyslipidemia, CVD). #Analyzed by 100ABSI. *Analyzed by 100TyG-ABSI. Numbers in bold represent *p* < 0.05.

### The predictive performance of obesity and lipid indexes for RKFD and progression to CKD

**Figure 2:**
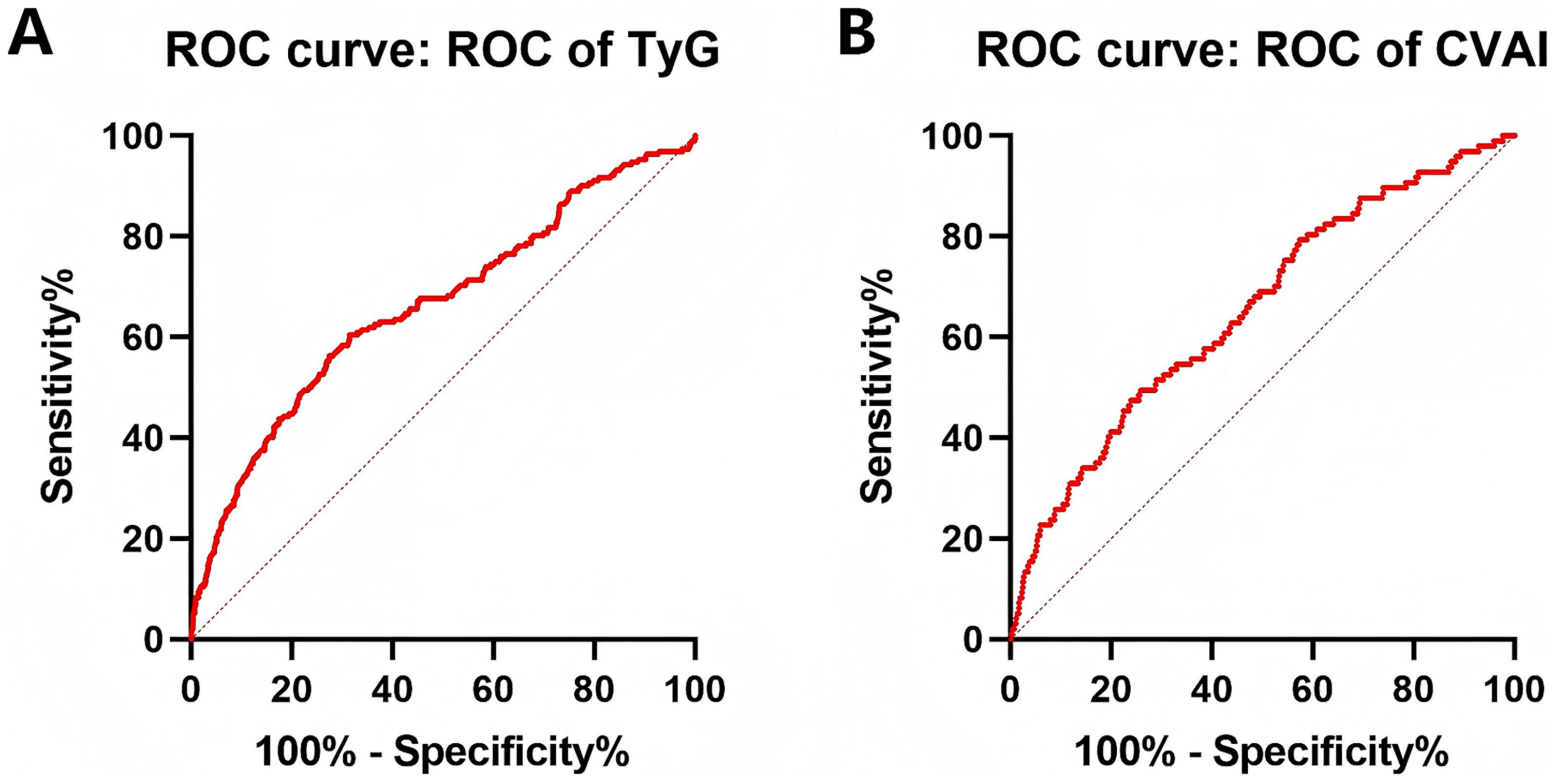
The predictive performance of TyG for RKFD **(A)** and CVAI for progression to CKD **(B)**.

To evaluate the predictive performance of obesity and lipid indexes for RKFD, we conducted ROC curve analysis using AUC metrics. Among these indexes, TyG had the highest AUC of [0.6635 (0.6201–0.7069)], followed sequentially by TyG-WWI [0.6629 (0.6210–0.7049)] and VAI [0.6628 (0.6200–0.7057)] (Table 4). In terms of the progression to CKD, CVAI had the highest AUC of [0.6542 (0.5976–0.7107)] (Table 5). Our study findings indicated that TyG was the most potent predictor for RKFD (Figure 2A), while CVAI stood out as the most predictive index for the progression to CKD among the obesity and lipid indexes (Figure 2B). The ROC curve graphs can be seen in Supplementary Figures S4, S5. In addition, we divided the participants into four groups based on the median values of TyG and CVAI, and compared whether there were differences in the incidence of RKFD or CKD among the groups. Regardless of the incidence of RKFD or CKD, participants with elevated TyG and CVAI levels showed a significant increase, while there were no significant differences among the other groups (Supplementary Table S1).

**Table 4:** ROC test of obesity and lipid indexes for RKFD.

Variables	AUC (95% CI)	Accuracy (95% CI)	Sensitivity (95% CI)	Specificity (95% CI)	PPV (95% CI)	NPV (95% CI)	Cutoff value	*p* value
BMI	0.5753 (0.5338–0.6169)	0.6686 (0.6534–0.6835)	0.6797 (0.6645–0.6948)	0.4583 (0.3879–0.5288)	0.9596 (0.9520–0.9672)	0.0702 (0.0561–0.0844)	24.81	<0.001
WC	0.6023 (0.5618–0.6427)	0.5947 (0.5789–0.6103)	0.5944 (0.5785–0.6104)	0.5990 (0.5296–0.6683)	0.9656 (0.9581–0.9732)	0.0723 (0.0596–0.0851)	87.15	<0.001
WHtR	0.6109 (0.5700–0.6518)	0.6490 (0.6336–0.6641)	0.6547 (0.6392–0.6701)	0.5417 (0.4712–0.6121)	0.9644 (0.9570–0.9717)	0.0765 (0.0623–0.0906)	0.564	<0.001
ABSI	0.5609 (0.5201–0.6018)	0.3082 (0.2936–0.3231)	0.2810 (0.2664–0.2956)	0.8229 (0.7689–0.8769)	0.9678 (0.9572–0.9784)	0.0570 (0.0484–0.0656)	0.08	0.0044
BRI	0.6109 (0.5700–0.6518)	0.6466 (0.6313–0.6618)	0.6519 (0.6364–0.6674)	0.5469 (0.4765–0.6173)	0.9646 (0.9573–0.9719)	0.0766 (0.0625–0.0907)	4.633	<0.001
C-index	0.5907 (0.5504–0.6310)	0.5477 (0.5317–0.5635)	0.5439 (0.5277–0.5600)	0.6198 (0.5511–0.6885)	0.9644 (0.9564–0.9724)	0.0669 (0.0553–0.0785)	1.294	<0.001
RFM	0.5739 (0.5297–0.6181)	0.7286 (0.7143–0.7427)	0.7448 (0.7307–0.7590)	0.4219 (0.3520–0.4917)	0.9606 (0.9535–0.9678)	0.0803 (0.0635–0.0970)	40.449	<0.001
WWI	0.5883 (0.5471–0.6295)	0.4847 (0.4688–0.5007)	0.4748 (0.4586–0.4911)	0.6719 (0.6055–0.7383)	0.9648 (0.9563–0.9733)	0.0633 (0.0527–0.0738)	11.068	<0.001
VAI	0.6628 (0.6200–0.7057)	0.7221 (0.7076–0.7363)	0.7316 (0.7172–0.7460)	0.5417 (0.4712–0.6121)	0.9680 (0.9614–0.9746)	0.0963 (0.0787–0.1139)	5.53	<0.001
CVAI	0.6298 (0.5887–0.6709)	0.5607 (0.5448–0.5765)	0.5554 (0.5393–0.5716)	0.6615 (0.5945–0.7284)	0.9688 (0.9614–0.9763)	0.0728 (0.0606–0.0850)	99.734	<0.001
LAP	0.6574 (0.6147–0.7000)	0.6446 (0.6292–0.6597)	0.6461 (0.6306–0.6617)	0.6146 (0.5457–0.6834)	0.9695 (0.9626–0.9763)	0.0840 (0.0695–0.0985)	37.061	<0.001
TyG	0.6635 (0.6201–0.7069)	0.6814 (0.6664–0.6961)	0.6855 (0.6704–0.7005)	0.6042 (0.5350–0.6733)	0.9704 (0.9639–0.9770)	0.0921 (0.0761–0.1080)	8.86	<0.001
TyG-BMI	0.6308 (0.5896–0.6720)	0.6937 (0.6788–0.7082)	0.7022 (0.6874–0.7171)	0.5312 (0.4607–0.6018)	0.9660 (0.9590–0.9729)	0.0861 (0.0701–0.1020)	220.5	<0.001
TyG-WC	0.6544 (0.6133–0.6955)	0.7065 (0.6917–0.7208)	0.7157 (0.7010–0.7304)	0.5312 (0.4607–0.6018)	0.9666 (0.9598–0.9734)	0.0898 (0.0732–0.1064)	799.654	<0.001
TyG-WHtR	0.6566 (0.6153–0.6978)	0.7239 (0.7095–0.7381)	0.7349 (0.7206–0.7493)	0.5156 (0.4449–0.5863)	0.9664 (0.9597–0.9731)	0.0931 (0.0757–0.1106)	5.115	<0.001
TyG-ABSI	0.6571 (0.6145–0.6996)	0.7579 (0.7440–0.7714)	0.7721 (0.7584–0.7857)	0.4896 (0.4189–0.5603)	0.9663 (0.9597–0.9728)	0.1018 (0.0823–0.1214)	0.768	<0.001
TyG-BRI	0.6360 (0.5951–0.6770)	0.6265 (0.6110–0.6419)	0.6283 (0.6126–0.6440)	0.5938 (0.5243–0.6632)	0.9670 (0.9598–0.9742)	0.0778 (0.0641–0.0915)	39.471	<0.001
TyG-RFM	0.6063 (0.5616–0.6510)	0.7819 (0.7685–0.7949)	0.8023 (0.7894–0.8153)	0.3958 (0.3267–0.4650)	0.9618 (0.9549–0.9686)	0.0956 (0.0752–0.1160)	364.881	<0.001
TyG-WWI	0.6629 (0.6210–0.7049)	0.6555 (0.6402–0.6706)	0.6574 (0.6420–0.6728)	0.6198 (0.5511–0.6885)	0.9704 (0.9637–0.9771)	0.0872 (0.0722–0.1021)	100.248	<0.001
TyG-CVAI	0.6457 (0.6043–0.6871)	0.7425 (0.7283–0.7563)	0.7564 (0.7424–0.7703)	0.4792 (0.4085–0.5498)	0.9649 (0.9582–0.9717)	0.0941 (0.0758–0.1124)	1,107.559	<0.001

CI, Confidence Interval; PPV, Positive predictive value; NPV, Negative predictive value.

**Table 5:** ROC test of obesity and lipid indexes for progression to CKD.

Variables	AUC (95% CI)	Accuracy (95% CI)	Sensitivity (95% CI)	Specificity (95% CI)	PPV (95% CI)	NPV (95% CI)	Cutoff value	*p* value
BMI	0.5420 (0.4816–0.6025)	0.4416 (0.4258–0.4575)	0.4357 (0.4198–0.4516)	0.6701 (0.5765–0.7637)	0.9807 (0.9741–0.9873)	0.0299 (0.0228–0.0371)	22.559	0.1569
WC	0.6089 (0.5492–0.6685)	0.4905 (0.4745–0.5064)	0.4850 (0.4690–0.5010)	0.7010 (0.6099–0.7921)	0.9842 (0.9785–0.9899)	0.0342 (0.0262–0.0422)	84.25	<0.001
WHtR	0.6178 (0.5582–0.6773)	0.6161 (0.6005–0.6315)	0.6174 (0.6018–0.6330)	0.5670 (0.4684–0.6656)	0.9821 (0.9767–0.9875)	0.0371 (0.0275–0.0467)	0.558	<0.001
ABSI	0.6278 (0.5723–0.6834)	0.7381 (0.7238–0.7519)	0.7454 (0.7315–0.7594)	0.4536 (0.3545–0.5527)	0.9813 (0.9763–0.9863)	0.0443 (0.0315–0.0571)	0.086	<0.001
BRI	0.6178 (0.5582–0.6773)	0.6150 (0.5994–0.6305)	0.6160 (0.6004–0.6316)	0.5773 (0.4790–0.6756)	0.9825 (0.9772–0.9878)	0.0376 (0.0279–0.0473)	4.508	<0.001
C-index	0.6427 (0.5852–0.7001)	0.6903 (0.6753–0.7049)	0.6937 (0.6789–0.7085)	0.5567 (0.4578–0.6556)	0.9837 (0.9788–0.9885)	0.0451 (0.0334–0.0569)	1.329	<0.001
RFM	0.5736 (0.5118–0.6354)	0.7182 (0.7037–0.7324)	0.7253 (0.7110–0.7397)	0.4433 (0.3444–0.5422)	0.9804 (0.9753–0.9856)	0.0403 (0.0285–0.0521)	40.149	0.0132
WWI	0.6336 (0.5761–0.6910)	0.6137 (0.5981–0.6292)	0.6136 (0.5980–0.6292)	0.6186 (0.5219–0.7152)	0.9841 (0.9790–0.9892)	0.0399 (0.0300–0.0499)	11.354	<0.001
VAI	0.6079 (0.5488–0.6669)	0.6242 (0.6086–0.6396)	0.6249 (0.6093–0.6404)	0.5979 (0.5004–0.6955)	0.9836 (0.9784–0.9887)	0.0398 (0.0297–0.0498)	4.404	<0.001
CVAI	0.6542 (0.5976–0.7107)	0.7383 (0.7241–0.7522)	0.7446 (0.7307–0.7586)	0.4948 (0.3953–0.5943)	0.9827 (0.9779–0.9875)	0.0480 (0.0347–0.0612)	123.542	<0.001
LAP	0.6161 (0.5568–0.6754)	0.5944 (0.5787–0.6100)	0.5932 (0.5775–0.6090)	0.6392 (0.5436–0.7347)	0.9844 (0.9793–0.9896)	0.0392 (0.0297–0.0488)	33.244	<0.001
TyG	0.5964 (0.5372–0.6557)	0.6158 (0.6002–0.6313)	0.6171 (0.6015–0.6327)	0.5670 (0.4684–0.6656)	0.9821 (0.9767–0.9875)	0.0371 (0.0275–0.0467)	8.76	0.0012
TyG-BMI	0.5707 (0.5073–0.6340)	0.6696 (0.6545–0.6845)	0.6742 (0.6591–0.6892)	0.4948 (0.3953–0.5943)	0.9809 (0.9756–0.9862)	0.0380 (0.0274–0.0485)	217.875	0.0173
TyG-WC	0.6182 (0.5576–0.6788)	0.6665 (0.6513–0.6814)	0.6699 (0.6548–0.6850)	0.5361 (0.4368–0.6353)	0.9823 (0.9772–0.9874)	0.0405 (0.0297–0.0513)	785.199	<0.001
TyG-WHtR	0.6242 (0.5633–0.6851)	0.6098 (0.5942–0.6253)	0.6096 (0.5939–0.6252)	0.6186 (0.5219–0.7152)	0.9840 (0.9789–0.9891)	0.0396 (0.0297–0.0494)	4.832	<0.001
TyG-ABSI	0.6514 (0.5960–0.7067)	0.4800 (0.4641–0.4960)	0.4727 (0.4567–0.4887)	0.7629 (0.6782–0.8475)	0.9871 (0.9819–0.9924)	0.0362 (0.0281–0.0443)	0.708	<0.001
TyG-BRI	0.6236 (0.5636–0.6835)	0.7412 (0.7270–0.7550)	0.7484 (0.7345–0.7623)	0.4639 (0.3647–0.5632)	0.9817 (0.9768–0.9866)	0.0457 (0.0327–0.0588)	44.916	<0.001
TyG-RFM	0.5821 (0.5211–0.6432)	0.8078 (0.7949–0.8202)	0.8202 (0.8079–0.8325)	0.3299 (0.2363–0.4235)	0.9792 (0.9742–0.9842)	0.0455 (0.0301–0.0609)	369.987	0.0057
TyG-WWI	0.6451 (0.5877–0.7026)	0.7508 (0.7368–0.7645)	0.7583 (0.7446–0.7720)	0.4639 (0.3647–0.5632)	0.9820 (0.9771–0.9868)	0.0475 (0.0340–0.0611)	103.923	<0.001
TyG-CVAI	0.6518 (0.5945–0.7091)	0.7448 (0.7307–0.7586)	0.7519 (0.7380–0.7657)	0.4742 (0.3749–0.5736)	0.9821 (0.9773–0.9870)	0.0473 (0.0340–0.0607)	1,108.644	<0.001

CI, Confidence Interval; PPV, Positive predictive value; NPV, Negative predictive value.

### Subgroup analysis

**Figure 3:**
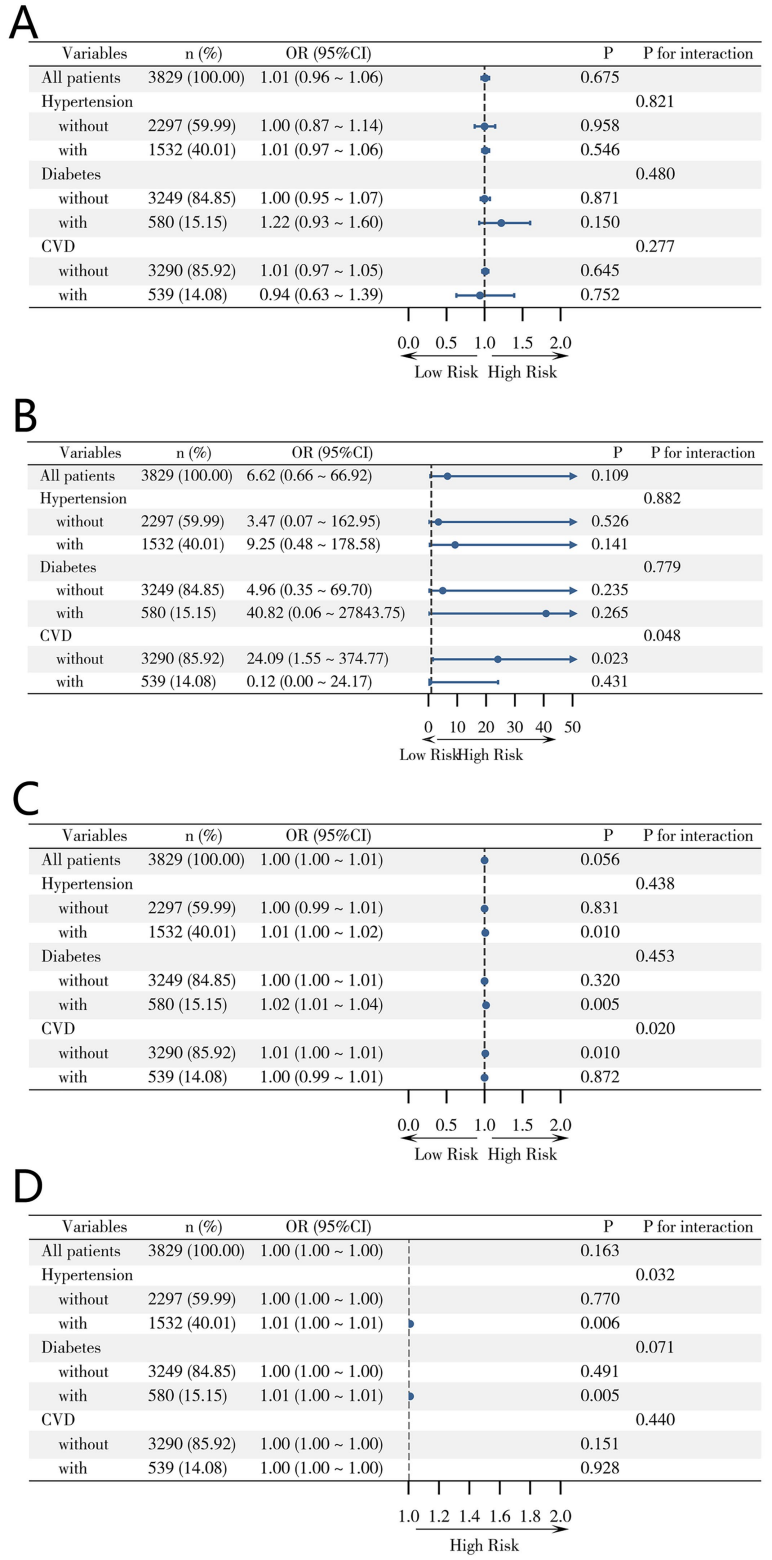
Subgroup analysis of C-index, RFM, TyG-RFM and TyG-CVAI. **(A)** subgroup analysis of C-index, **(B)** subgroup analysis of RFM, **(C)** subgroup analysis of TyG-RFM, **(D)** subgroup analysis of TyG-CVAI.

We further investigated the relationships between obesity and lipid indexes and RKFD (Supplementary Figure S6) or progression to CKD (Supplementary Figure S7) in subgroups stratified by hypertension (with or without), diabetes (with or without) and CVD (with or without). In terms of the association between obesity and lipid indexes and RKFD, there was no significant difference between populations with hypertension, diabetes, or CVD and those without. C-index, RFM and TyG-RFM showed association with progression to CKD in people without CVD, which was not present in analyses for people with CVD and the population as a whole (Figures 3A–C). In addition, higher TyG-CVAI promotes progression to CKD in people with hypertension (Figure 3D).

## Discussion

In this prospective, longitudinal, cohort study, we investigated the relationship between 20 obesity and lipid indexes and RKFD and CKD in Chinese adults. We found that 11 indexes (WC, VAI, CVAI, LAP, TyG, TyG-WC, TyG-WHtR, TyG-ABSI, TyG-RFM, TyG-WWI and TyG-CVAI) were independent risk factors for RKFD, and TyG had the most predictive value of all the indexes for RKFD. Additionally, WC, LAP, TyG-WC and TyG-ABSI were found to be associated with CKD after adjustment for all confounding factors, whereas CVAI exhibited the highest predictive value for CKD. The results of this study were consistent with the results of previous studies (24–27).

.We found that participants with a higher baseline eGFR were more prone to experience RKFD during the follow-up period. In this study, the incidence of RFKD was 7.46% in participants with eGFR greater than 90 mL/min/1.73 m^2^, whereas the incidence in participants with eGFR less than 90 mL/min/1.73 m^2^ was only 2.35%. This phenomenon may reflect that in individuals with relatively better kidney function, certain underlying pathophysiological mechanisms are more likely to trigger a sharp deterioration in renal function. This result is consistent with some previous research findings (28, 29). This might be related to the overactivation of renal function compensation mechanisms, such as a hyperfiltration state of the glomeruli, or it could be associated with certain biomarkers or genetic factors that have not yet been clearly identified. These studies suggest that when assessing the risk of RKFD, reliance on baseline renal function levels alone is insufficient; other clinical and biological indicators should also be taken into account.

In this study, TyG had the strongest predictive power among all the indexes associated with RKFD. TyG is an alternative biomarker for insulin resistance (IR) (30), which has been proven to be associated with renal function decline in the elderly (31, 32). IR can directly affect renal function by promoting glomerular ultrafiltration, impairing endothelial function and increasing vascular permeability. In addition, IR-induced blood glucose and dyslipidemia can lead to renal fibrosis and tubular damage, resulting in decreased renal function (32, 33). This may be a potential reason to explain the significant association between TyG, which was calculated with TG and FPG, and RKFD. Participants with higher blood glucose levels or those taking diabetes medication had significantly higher TyG values than other participants, and they had a higher probability of developing RKFD. However, the predictive power of TyG for RKFD was not related to whether the participants have diabetes. This indicates that TyG, as a strong predictive factor for RKFD, is only related to islet function and is not affected by other diabetic complications such as microinflammation or microcirculatory disorders. Notably, there was a significant association between WC and RKFD, and WHtR and BRI calculated based on WC and height showed a nonlinear relationship with RKFD. WC is closely associated with the accumulation of visceral fat as an indicator of abdominal obesity. A Japanese study confirmed that WC significantly affects the progression of kidney injury, and maintaining a normal range of WC can reduce the risk of kidney injury (34). The association between increased WC and decreased eGFR may be mediated by increased renal vascular resistance and correlated with increased IR levels (35). Central obesity can be better defined by WC and the indices of correlation of WC than by BMI. Central obesity is more closely related to metabolic derangements (36). This may explain the “obesity paradox” of CKD, where people with higher BMI have a lower risk of declining eGFR (37).

Obesity is an independent risk factor for CKD, and the metabolic disorders it causes have been confirmed as the etiology of CKD (38). Our study revealed that WC, LAP, TyG-WC and TyG-ABSI exhibit associations with CKD, and confirmed that the CVAI was outperforming other indexes in forecasting CKD. LAP, TyG-WC and TyG-ABSI are all indices calculated based on WC. This once again verifies the important impact of WC and central obesity on CKD. Particularly noteworthy was the CVAI, a visceral fat assessment tool specifically developed for the Chinese population, showed significant potential as a unique and effective metabolic indicator for assessing visceral fat. Gao et al. found that CVAI was negatively correlated with eGFR and individuals with diabetes exhibited a higher susceptibility to CKD development associated with elevated CVAI levels (39). Compared to the other obesity and lipid indexes, CVAI incorporates multiple parameters, including BMI, WC, TG, and HDL-C, reflecting increased ectopic fat depots and providing a more comprehensive depiction of abnormal lipid metabolism. Visceral fat is a major driver of systemic inflammation, as it produces more pro-inflammatory factors than subcutaneous fat (39). Pro-inflammatory factors and adipokines induce oxidative stress and inflammation, and even lead to kidney fibrosis, which ultimately impairs kidney function (11). Therefore, we believe that invasive anthropometric indexes that incorporate lipid metabolism parameters provide a more comprehensive picture of human obesity than non-invasive anthropometric indexes.

This study provides clinicians and researchers with valuable insights into predicting the incidence of RKFD and CKD. The results of this study are useful for the rapid assessment and risk prediction of kidney function in the field of public health. Clinicians can use indicators such as TyG and CVAI for early screening of high-risk populations for kidney disease. This helps achieve early detection and early treatment of kidney disease and prevent deterioration of kidney function. However, it should be pointed out that renal function damage is a complex process involving multiple factors of the whole body. Therefore, in clinical practice, clinicians should comprehensively consider the specific situation of patients and select appropriate obesity and lipid indices for renal function monitoring and CKD risk assessment, so as to provide patients with more effective prevention and treatment strategies.

## Strengths and limitations

There are some strengths in this study. First, the data used in this study were from CHARLS, a nationwide study with broad coverage and a long follow-up period, which has strong data credibility. Second, to reduce the influence of muscle mass on the level of renal function in middle-aged and elderly people, we calculated eGFR by combining creatinine and cystatin C. Third, we selected 20 obesity and lipid indexes for analysis, so as to more comprehensively evaluate the association between obesity and renal function.

However, there are some limitations to this study. First, this study only used data from middle-aged and elderly people in China, which made it difficult to extrapolate the research results to people of different races and ages. Second, the data in this study did not have urine protein test results, which caused the incidence of CKD to be underestimated; at the same time, it was not rigorous enough to diagnose and define CKD through one eGFR result. Third, the removal of participants due to missing data may result in selection bias in the results.

## Conclusion

We proved multiple obesity and lipid indexes are prospectively associated with the incidence of RKFD and CKD in Chinese with normal kidney function over age 45. Among all the indexes, TyG has the largest predictive value for RKFD, while CVAI has the largest predicted value for CKD. Compared with non-invasive anthropometric indexes, invasive anthropometric indexes have a more significant association with RKFD and CKD and have higher predictive value.

## Data Availability

Publicly available datasets were analyzed in this study. This data can be found at: http://charls.pku.edu.cn/index.htm.

## Footnotes

1
http://charls.pku.edu.cn/

## Glossary

CKD: Chronic kidney disease
eGFR: Estimated glomerular filtration rate
CVD: Cardiovascular diseases
BMI: Body mass index
WC: Waist circumference
WHtR: Waist-to-height ratio
ABSI: A body shape index
BRI: Body roundness index
C-index: Conicity index
RFM: Relative fat mass
WWI: Weight-adjusted waist index
VAI: Visceral adiposity index
CVAI: Chinese visceral adiposity index
LAP: Lipid accumulation product
TyG: Triglyceride glucose
RKFD: Rapid kidney function decline
CHARLS: Chinese Longitudinal Study of Health and Retirement
TG: triglyceride
HDL-C: High-density lipoprotein cholesterol
FBG: Fasting blood glucose
HbA1c: Glycosylated Hemoglobin, Type A1C
SBP: Systolic blood pressure
DBP: Diastolic blood pressure
TC: Total cholesterol
UA: Uric acid
CRP: C reactive protein
SD: Standard deviation
OR: Odds ratio
CI: Confidence interval
RCS: Restricted cubic spline
ROC: Receiver operating characteristic
AUC: Area under the curve
