# Supplementary figures and images for: Association of obesity and lipid indexes with rapid kidney function decline and the progression to chronic kidney disease: a study from a large longitudinal cohort among middle-aged and older adults in China
Source: Front Med (Lausanne). 2026 May 4;13:1816603. doi: 10.3389/fmed.2026.1816603 (PMC13213437; doi:10.3389/fmed.2026.1816603)

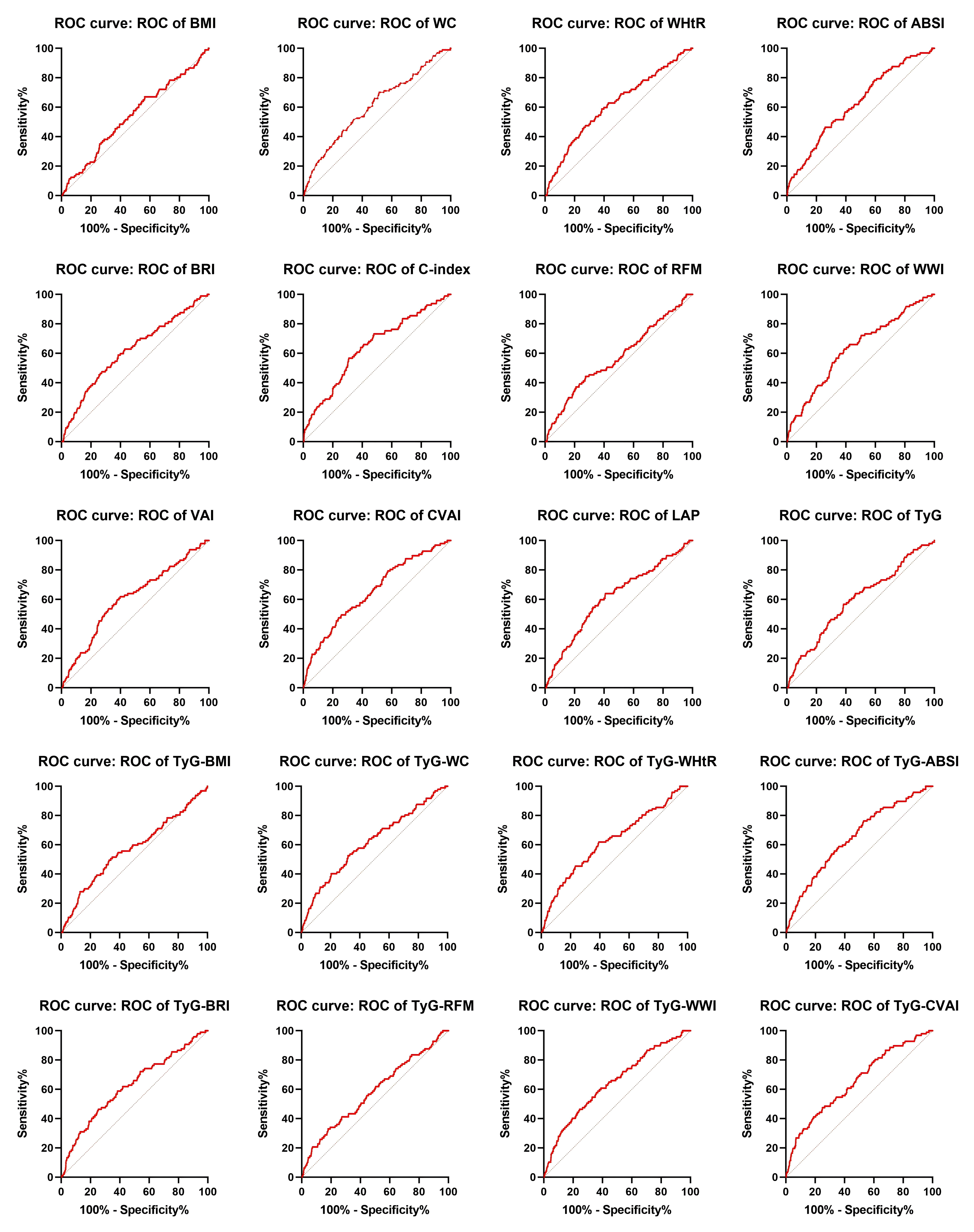

Supplement: Supplementary file 3 [file Image_3.PNG]

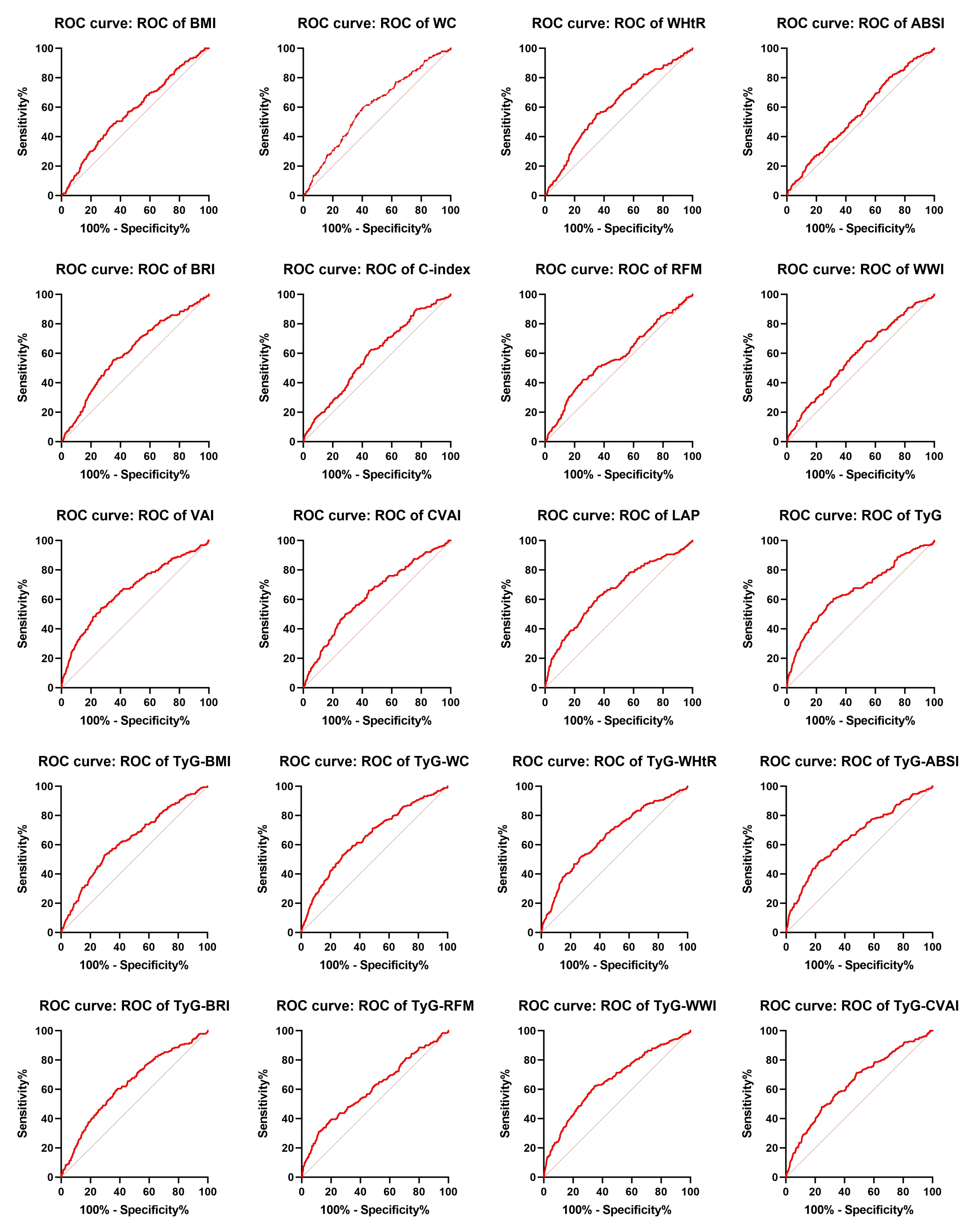

Supplement: Supplementary file 4 [file Image_4.PNG]

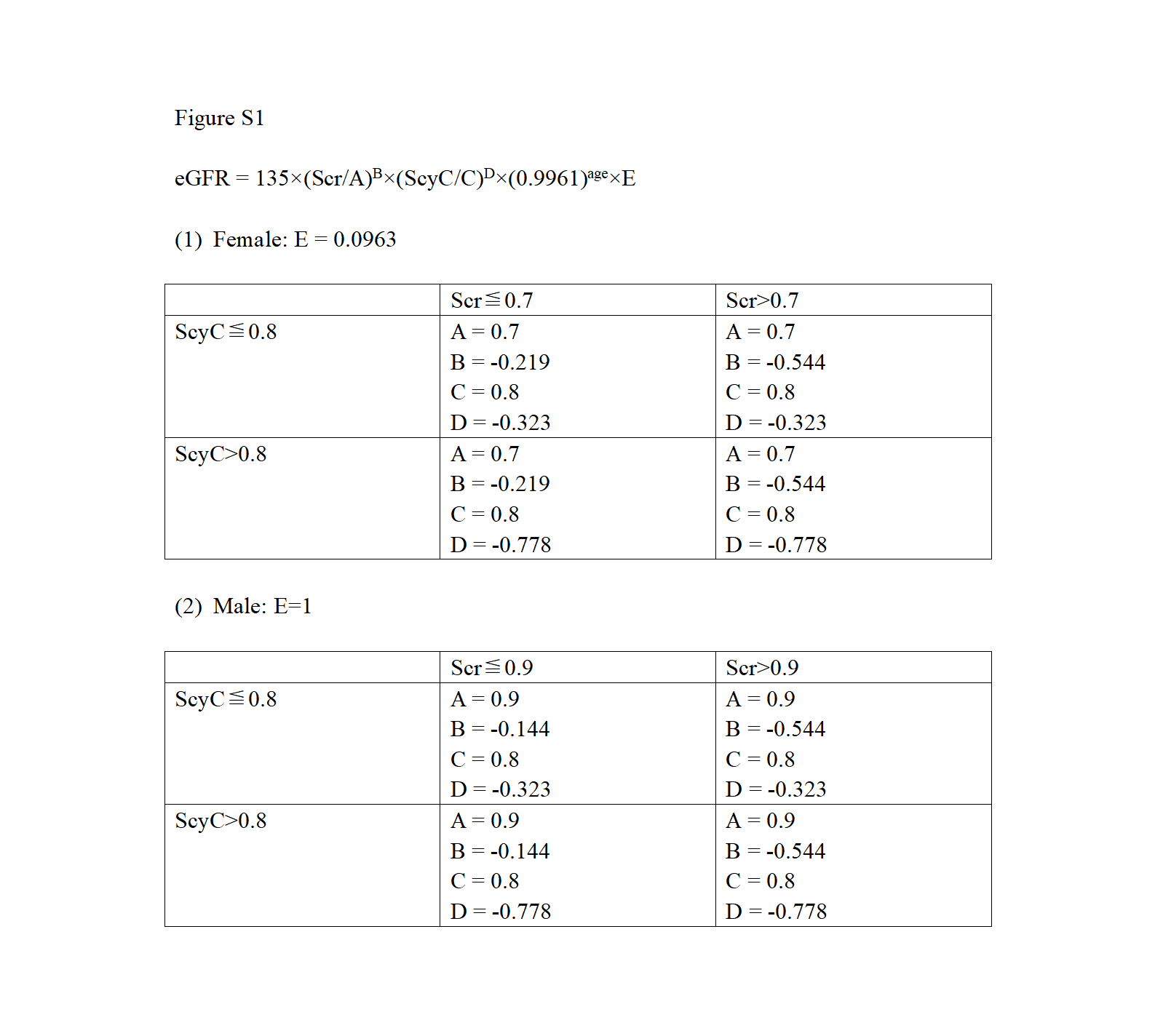

Supplement: Supplementary file 5 [file Image_5.PNG]
