# Supplementary material for: Association of obesity and lipid indexes with rapid kidney function decline and the progression to chronic kidney disease: a study from a large longitudinal cohort among middle-aged and older adults in China
Source: Front Med (Lausanne). 2026 May 4;13:1816603. doi: 10.3389/fmed.2026.1816603 (PMC13213437; doi:10.3389/fmed.2026.1816603)
Supplement: Supplementary file 8 [file Table_1.DOCX]

**Table S1**

|  | **RKFD** | **CKD** | **Total** |
| --- | --- | --- | --- |
| **A** | 109 | 53 | 1352 |
| **B** | 21^#^ | 10 | 562 |
| **C** | 23^#^ | 14 | 562 |
| **D** | 39^#^ | 20^#^ | 1353 |
| **Total** | 192 | 97 | 3829 |

A: TyG > 8.57364 & CVAI > 95.35553

B: TyG > 8.57364 & CVAI < 95.35553

C: TyG < 8.57364 & CVAI > 95.35553

D: TyG < 8.57364 & CVAI < 95.35553

#: There was statistical difference compared with Group A, *p* < 0.05.
